# How far has diabetes‐related foot disease research progressed in Australia? A bibliometric review (1970–2023)

**DOI:** 10.1002/jfa2.12012

**Published:** 2024-04-16

**Authors:** Peta E. Tehan, Byron M. Perrin, Peter A. Lazzarini, Ibrahim S. Al‐Busaidi, Matthew R. Carroll

**Affiliations:** ^1^ Department of Surgery School of Clinical Sciences Monash University Clayton Victoria Australia; ^2^ Department of Rural Health Sciences La Trobe Rural Health School Bendigo Victoria Australia; ^3^ Allied Health Research Collaborative The Prince Charles Hospital Brisbane Queensland Australia; ^4^ School of Public Health and Social Work Queensland University of Technology Brisbane Queensland Australia; ^5^ Department of Primary Care and Clinical Simulation University of Otago Christchurch New Zealand; ^6^ Department of Podiatry School of Clinical Sciences Faculty of Health and Environmental Sciences Auckland University of Technology Auckland New Zealand

**Keywords:** Australia, bibliometric analysis, diabetes mellitus, diabetes‐related foot disease, diabetic foot, research

## Abstract

**Background:**

Diabetes‐related foot disease (DFD) is a leading cause of the Australian and global disease burdens and requires proportionate volumes of research to address. Bibliometric analyses are rigorous methods for exploring total research publications in a field to help identify volume trends, gaps and emerging areas of need. This bibliometric review aimed to explore the volume, authors, institutions, journals, collaborating countries, research types and funding sources of Australian publications investigating DFD over 50 years.

**Methods:**

A systematic search of the Scopus^®^ database was conducted by two independent authors to identify all Australian DFD literature published between 1970 and 2023. Bibliometric meta‐data were extracted from Scopus^®^, analyzed in Biblioshiny, an R Statistical Software interface, and publication volumes, authors, institutions, journals and collaborative countries were described. Publications were also categorised for research type and funding source.

**Results:**

Overall, 332 eligible publications were included. Publication volume increased steadily over time, with largest volumes (78%) and a 7‐fold increase over the last decade. Mean co‐authors per publication was 5.6, mean journal impact factor was 2.9 and median citation was 9 (IQR2‐24). Most frequent authors were Peter Lazzarini (14%), Vivienne Chuter (8%) and Jonathon Golledge (7%). Most frequent institutions affiliated were Queensland University Technology (33%), University Sydney (30%) and James Cook University (25%). Most frequent journals published in were Journal Foot and Ankle Research (17%), Diabetic Medicine (7%), Journal Diabetes and its Complications (4%) and International Wound Journal (4%). Most frequent collaborating countries were the United Kingdom (9%), the Netherlands (6%) and the United States (5%). Leading research types were etiology (38%), treatment evaluation (25%) and health services research (13%). Leading funding sources were no funding (60%), internal institution (16%) and industry/philanthropic/international (10%).

**Conclusions:**

Australian DFD research increased steadily until more dramatic increases were seen over the past decade. Most research received no funding and mainly investigated etiology, existing treatments or health services. Australian DFD researchers appear to be very productive, particularly in recent times, despite minimal funding indicating their resilience. However, if the field is to continue to rapidly grow and address the very large national DFD burden, much more research funding is needed in Australia, especially targeting prevention and clinical trials of new treatments in DFD.

## 1 BACKGROUND

Diabetes is the most rapidly growing leading cause of the global burden of disease, affecting an estimated 527 million people worldwide [1, 2]. Diabetes‐related foot disease (DFD) is the leading cause of global diabetes‐related hospitalisations, amputations and burden of disease, affecting an estimated 20 million people worldwide with an additional 130 million at risk [3, 4]. DFD is defined as infection, ulceration or tissue destruction of the foot in a person with diabetes, underpinned by peripheral neuropathy and/or peripheral artery disease [5, 6, 7].

Australia has been reported to have higher DFD‐related hospitalisation and amputation rates than similar countries [7, 8, 9]. However, substantial variation exists between different geographical areas within Australia and also within indigenous populations [10, 11, 12]. It is estimated that on any given day in Australia, 50,000 people have DFD, 1000 are in hospital due to DFD, 12 will undergo a DFD‐related amputation and four people will die due to DFD [13, 14]. It is currently estimated that 1%–2% of healthcare expenditure in Australia and globally is related to DFD, yet research into DFD attracts less than 0.01% of competitive research funding [14, 15, 16, 17]. This disparity highlights the lack of focus on this major health problem 17 and greater research contributions in DFD are required to address this large burden.

It is also important to understand the institutions, researchers, research type and level of funding that mostly produce research in a field, such as DFD [18, 19, 20, 21]. For example, it has been reported that there is a gross disparity in the level of research funding for DFD compared to other conditions with similar burdens [14, 16]. However, it is not clear how the research type and level of funding is distributed for DFD research in Australia. Understanding the research taking place in Australia and the means by which it receives funding is important to help Australian researchers and funders prioritise research for consumers, clinicians, funders and peak bodies. This, in turn, should improve the allocation of funds to the research prioritised at addressing existing gaps in the evidence base that targets reductions in the burden of disease for individuals with DFD and the nation.

Significant advances in the understanding of DFD have been made through the conduct of research internationally [22, 23]. Recent international bibliometric analyses have demonstrated that the global volume of research in DFD is increasing [18, 19, 20, 21]. While Australia is in the top 10 countries for total DFD research volume in these global bibliometric analyses [18, 20, 23], the trends in terms of volume, authors, institutions, journals, collaborating countries, research types and funding sources that drive this research within Australia have not been reported. In addition, it is unclear whether the research produced by Australian researchers targets priority areas for DFD stakeholders (including people with DFD) [17], or are aligned with international recommendations [24]. Therefore, the objective of this study was to conduct a bibliometric review that aimed to explore the volume, authors, institutions, journals, collaborating countries, research types and funding sources of Australian research publications investigating DFD over 50 years.

## 2 METHOD

This was a bibliometric analysis of Australian publications investigating DFD between January 1970 and November 2023 using data from the Scopus^®^ database (Elsevier, Amsterdam, the Netherlands). We chose the Scopus^®^ database for several reasons. Firstly, Scopus^®^ currently has the largest abstract and citation database for research literature [25]. Secondly, it allows searching by publication, author or affiliation, with the ability to refine results by access type, source type, year, language, author, affiliation and funding sponsor. Thirdly, it has over 71.2 million records post 1969, includes a broader range of journals than PubMed and Web of Science and its citation analysis is faster and includes more publications than the citation analysis of Web of Science [26].

### 2.1 Search strategy

**FIGURE 1:**
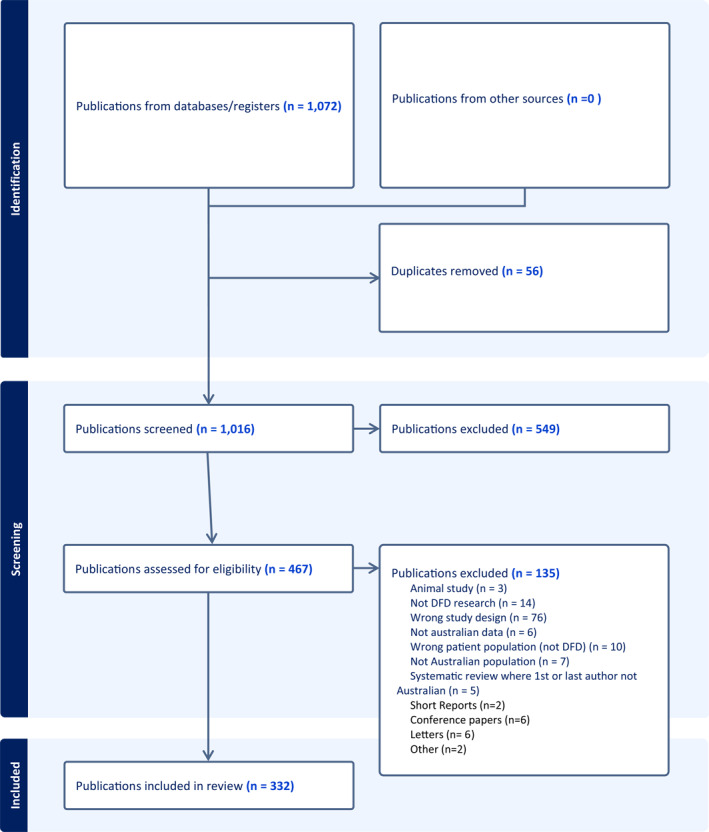
PRISMA flowchart.

The Scopus search strategy terms were developed based on previous bibliometric research on DFD and are presented in Table 1 [19]. The search and retrieval process are shown in the PRISMA flow diagram (Figure 1).

**TABLE 1:** Scopus^®^ search strategy.

Keywords	
1. Diabet*	
2. Peripheral neuropathy	
3. Peripheral arterial	
4. Amputation	
5. Infect*	
6. Ulcer	
7. Charcot neuro*	
8. Foot	
9. Feet	
Search strategy	
1 = 1	
2 = 2 OR 3 OR 4 OR 5 OR 6 OR 7 OR 8 OR 9	
3 = 1 AND 2	
Search restrictions	
Year	1970–2023
Language	English
Source	Article
Author affiliation	Australian

### 2.2 Study selection

The titles and abstracts of all identified publications were downloaded from the Scopus^®^ database and exported into the online systematic review application Covidence^©^ (Veritas Health Innovation, Melbourne, Australia). Titles, abstracts and full text review were independently screened by two authors (PT and MC). Publications were included if they were original articles or systematic reviews, conducted by an Australian Institution and in Australian population, had at least one author with an Australian affiliation and were published in English. Publications were included if the field of research was related to DFD [6, 24]. This included diabetes‐related peripheral neuropathy, Charcot neuroarthropathy, peripheral artery disease, ulceration, infection and/or amputation. Publications were excluded if they were case reports, commentaries, conference abstracts, research letters, guidelines or editorials. Any conflicts were discussed between two authors (PT and MC) until consensus was reached. Additional publications were identified by backward snowballing checks of reference lists [27].

### 2.3 Data extraction

Metadata of all included publications were extracted from Scopus and imported into Biblioshiny^®^ (based on R version 3.6.1, Bibliometrix package version 2.2.1; University of Naples Federico II, Naples, Italy, 2016)[28] for data extraction. The following bibliometric indicators were extracted from each publication: year of publication, journal name, journal impact factor (IF) (in year prior to publication, 2023 using the Web of Science Journal Citation Reports™ tool [Clarivate Analytics, Philadelphia, Pennsylvania, USA]), number of citations (determined by the Scopus^®^ database [Elsevier, Amsterdam, Netherlands]), author names, total authors per manuscript and institutional and country affiliation of each author.

### 2.4 Data synthesis

To investigate the areas of research focus, publications were categorised according to the research type dimension of the United Kingdom Clinical Research Commission (UKCRC) Health Research Classification System by one author (BMP) [29]. This widely used system allows the classification of all types of health‐related research from basic to applied research [29]. This system classifies types of research activities using 48 codes within eight overarching groups: (1) underpinning research; (2) etiology; (3) prevention of diseases and conditions; (4) detection, screening and diagnosis; (5) development of treatments; (6) evaluation of treatments (7) management of diseases and conditions; and (8) health services research. The two‐digit research activity underpinning sub‐code was also applied (48 total codes), which provided greater detail on the specific research activity.

To investigate funding sources, publications were categorised according to the definitions outlined in the Australian Government Higher Education Research Data Collection specifications [30], based on the funding details and text provided for each publication in Scopus by one author (PAL). This system classifies funding categories into six groups: (1) Australian competitive grants (such as National Health and Medical Research Council [NHMRC]); (2) Other public sector funding (such as state government funding); (3) Industry and other sources (including private sector and philanthropic organisations); (4) Cooperative Research Centre (CRC) funding; (5) Internal institution funding of the authors; and (6) Nil funding received or reported. In cases where a publication qualified for multiple categories, the highest‐level category was assigned. For instance, if a publication met the criteria for categories 1, 4, and 5, it would be recorded only under category 1. For descriptive analyses, means were calculated within Biblioshiny ^®^ software, with median and interquartile ranges calculated in Excel (Microsoft Corporation, 2018). Research type and funding were each categorised individually by members of the research team (B.M.P research type and P.L research funding). To assess interrater reliability of research type and funding categorisations, a third researcher (P.T) who was blinded to the original categorisations assessed a random sample of 30 publications. Interrater reliability was assessed by calculating Cohen's κ‐coefficient.

## 3 RESULTS

### 3.1 Descriptive findings

**FIGURE 2:**
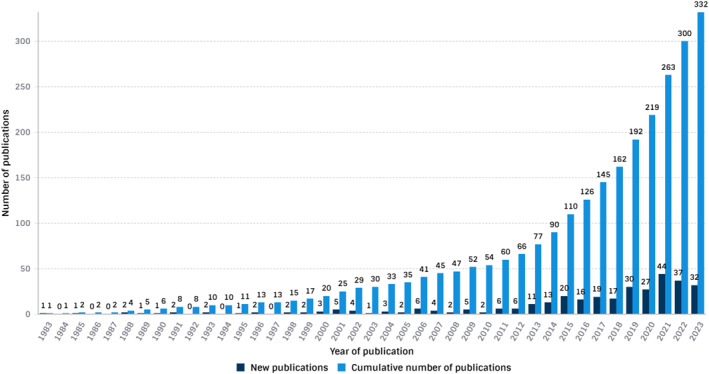
Annual number of Australian DFD publications (1970–2022). DFD, diabetes‐related foot disease.

Table 2 presents the publication characteristics of a total of 332 Australian publications related to DFD that were deemed eligible and included (Figure 1, Supporting Information S1). Figure 2 shows the schematic trend of Australian produced DFD publications increased steadily from the first identified publication in 1983 (Figure 2) until 2012, and then increased exponentially with a five‐fold increase in annual publications from 2012 to 2021, with the highest output in 2021 (15%) and a total of 266 (68%) of publications occurring in the past decade. The total publications had a total of 6075 citations, with a mean citation rate of 5.6 per publication (median: 9, IQR 2–24, range 0–151), including 47 (14%) that were not yet cited. The median journal impact factor of the journals in which the publications were published was 2.9 (IQR: 2.4–4.1, range 0.29–16.2).

**TABLE 2:** Publication characteristics.

Total number of articles	332
Original articles	288
Systematic reviews	44
Mean years from publication	8
Mean citations per year per article	18.19
Citations	6075
Total authors	1025
Mean co‐authors per article	5.6
International co‐authorships (%)	29.3
Single‐authored publications	3

### 3.2 Most frequent authors, institutions, journals and collaborating countries

**FIGURE 3:**
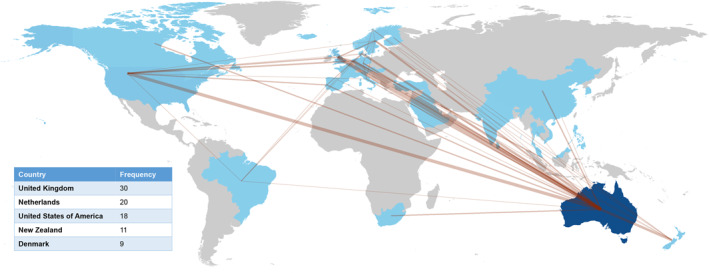
Top 5 International collaboration countries.

There were a total of 1025 unique authors from the 332 publications for a mean of 5.6 co‐authors per publication. Table 3 displays the most frequent authors of the publications were Peter Lazzarini (14%), Vivienne Chuter (8%), and Jonathon Golledge (7%). Table 4 displays the most frequent institutional affiliations were the Queensland University of Technology (QUT) (33%), University of Sydney (30%) and James Cook University (25%). In 324 of the included publications, an Australian institution was the primary affiliation. Nine publications included one author with an Australian affiliation. Table 5 displays the most frequent journals in which publications were published, which were the Journal of Foot and Ankle Research (17%), Diabetic Medicine (7%), International Wound Journal and Journal of Diabetes and its Complications (both 4%). Figure 3 presents the five most common countries in which research collaboration occurred including the United Kingdom (9%), followed by the Netherlands (6%) and the United States of America (5%).

**TABLE 3:** Top 10 most frequent authors of Australian DFD publications.

Author	Articles *n*, (%)
Lazzarini PA	48 (14)
Chuter VH	27 (8)
Golledge J	24 (7)
Fernando ME	21 (6)
Malone M	19 (6)
van Netten JJ	18 (5)
Twigg SM	14 (4)
Yue DK	12 (4)
Dickson HG	12 (4)
Jensen SO	11 (3)
Hamilton EJ	11 (3)
Perrin BM	11 (3)
Yue DK	11 (3)

Abbreviation: DFD, diabetes‐related foot disease.

**TABLE 4:** Top 10 most frequent institutions of Australian DFD publications.

Institution	Articles *n*, (%)
Queensland University of Technology	109 (33)
University of Sydney	98 (30)
James Cook University	84 (25)
University of Newcastle	79 (24)
University of Western Australia	63 (19)
Western Sydney University	61 (18)
La Trobe University	49 (15)
University of Adelaide	28 (8)
Royal Melbourne Hospital	28 (8)
Fiona Stanley Hospital	26 (8)

Abbreviation: DFD, diabetes‐related foot disease.

**TABLE 5:** Top 10 most frequent journals publishing Australian DFD publications.

Sources	*n* (%)	Quartile	IF (2022/3)
Journal of foot and ankle research	57 (17)	2	3.05
Diabetic medicine	23 (7)	1	4.21
Journal of diabetes and its complications	15 (4)	1	3.21
International wound journal	15 (4)	1	3.10
Diabetes research and clinical practice	11 (3)	1	5.1
PLOS one	10 (3)	1	3.7
Diabetes/metabolism research and reviews	10 (3)	1	8.0
European journal of vascular and endovascular surgery	8 (2)	1	5.33
Clinical biomechanics	7 (2)	3	1.8
Diabetes care	6 (2)	1	16.2

Abbreviations: DFD, diabetes‐related foot disease; IF, impact factor.

### 3.3 Most cited publications

Table 6 presents the most highly cited publications, which were a Cochrane systematic review by Bergin and Wraight [31] (*n* = 151) and two original research papers by Delbridge et al. [32] (*n* = 144) and Tapp et al. [33] (*n* = 135).

**TABLE 6:** Top 10 cited Australian DFD publications.

Authors (year)	Publication title	Journal	Citations (*n*)	Average citations per year
Bergin & Wraight (2006)	Silver‐based wound dressings and topical agents for treating diabetic foot ulcers.	*Cochrane Database of Systematic Reviews*	151	7.95
Delbridge et al. (1988)	Limited joint mobility in the diabetic foot: relationship to neuropathic ulceration.	*Diabetic Medicine*	144	3.89
Tapp et al, (2003)	Foot complications in type 2 diabetes: An Australian population‐based study.	*Diabetic Medicine*	135	6.14
Barth et al. (1991)	Intensive education improves knowledge, compliance, and foot problems in type 2 diabetes.	*Diabetic Medicine*	134	3.94
Fernando et al. (2013)	Biomechanical characteristics of peripheral diabetic neuropathy: A systematic review and meta‐analysis of findings from the gait cycle, muscle activity and dynamic barefoot plantar pressure.	*Clinical Biomechanics*	128	10.67
Davis et al (2006)	Predictors, consequences and costs of diabetes‐related lower extremity amputation complicating type 2 diabetes: The fremantle diabetes study.	*Diabetologia*	126	6.63
Rogers et al (2001)	Passive tactile sensory input improves stability during standing	*Experimental Brain Research*	126	5.25
Zhang et al (2020)	Global disability burdens of diabetes‐related lower‐extremity complications in 1990 and 2016	*Diabetes Care*	112	22.40
Xu et al (2007)	Bacterial load predicts healing rate in neuropathic diabetic foot ulcers	*Diabetes Care*	87	4.83
Behrendt et al (2018)	International variations in amputation practice: a VASCUNET report	*European Journal of Vascular & Endovascular Surgery*	86	12.29

Abbreviation: DFD, diabetes‐related foot disease.

### 3.4 Leading research types

**FIGURE 4:**
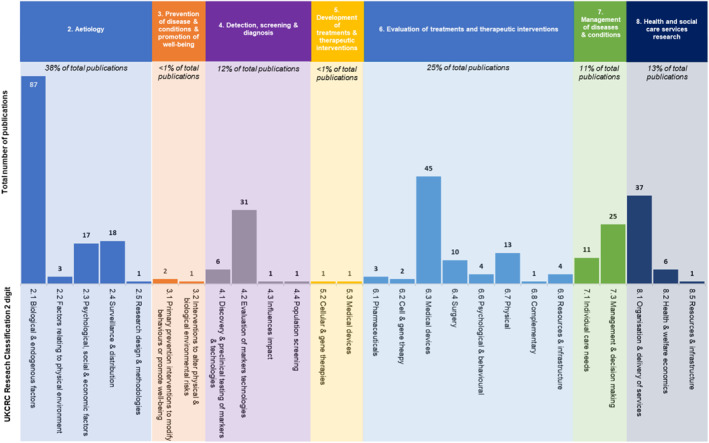
Research type for DFD Australian DFD publications categorized by the UKCRC Health Research Classification System. DFD, diabetes‐related foot disease.

Figure 4 shows the proportion of publications categorised with the UKCRC Health Research Classification System, according to the one‐digit overarching code groups and the two‐digit codes, respectively. The research category with most Australian DFD publications were etiology (38%), evaluation of treatments (25%), health services research (13%), detection, screening and diagnosis (12%), and management of disease and conditions (11%). Very few publications were related to prevention of diseases and conditions (<1.0%) and development of treatments and therapeutic interventions (<1%). The interrater reliability of research type categorisation was 0.91, indicating excellent agreement.

### 3.5 Leading funding sources

Table 7 shows the proportion of publications categorised by the Australian Government's categories of research funding received. Most publications reported no research funding (60%), with only some reporting internal institution (16%), industry/philanthropic/international (10%), Australian competitive grant (7%), other public sector (5%) and CRC (<1%) funding. The interrater reliability of research funding categorisation was 0.91, indicating excellent agreement.

**TABLE 7:** Funding sources of Australian DFD publications.

Funding category^a^	*n* (%)
Nil reported	200 (60)
Australian competitive grants	24 (7)
Other public sector	17 (5)
Industry/philanthropic/international	35 (10)
Cooperative research centre	2 (<1)
Internal institutional	54 (16)

Abbreviation: DFD, diabetes‐related foot disease.

^a^ Based on the definitions in the Australian Government Higher Education Research Data Collection.

## 4 DISCUSSION

Bibliometric analyses are an effective and rigorous method to describe current research trends and identify current gaps in research. The results of the current study described a steady increase in the total number of Australian DFD publications with a dramatic seven‐fold increase over the past decade. The authors with the highest contributions were Peter Lazzarini, Vivienne Chuter and Jon Golledge. The highest institutional output was Queensland University of Technology, followed by the University of Sydney and James Cook University. Etiology was the most frequently published research type, followed by treatment evaluation and health services research. The majority of research did not report any funding sources.

The substantial increase in Australian DFD research over the past 10 years is consistent with the DFD research reported internationally and in other nations, including New Zealand and Gulf countries [19, 20, 21, 23]. Previous global DFD bibliometric analyses have demonstrated that publications have more than doubled worldwide from 2007 to 2017 [20], and tripled from 2010 to 2020 [23], with the United States of America accounting for approximately one third of all publications [20] However, there was a seven‐fold increase in our findings indicating an increased attention to the DFD field by researchers in Australia in particular. Other factors that may have also influenced the increase in output include the work and role of international and national research bodies advocating for DFD, such as the International Working Group for the Diabetic Foot (IWGDF) and the establishment of Diabetes Feet Australia. In other broad chronic health conditions such as gout and osteoarthritis, there have been similar upward trajectories in increasing output in recent times; however, a much greater overall volume of scholarly output was also seen in these other conditions compared to DFD research [34, 35].

Citation frequency is commonly used as a quality indicator of published research and a marker of research impact in bibliometric analysis [19, 20, 21, 34]. Factors that may influence citation frequency include perceived quality of the research publication, quality of journal, relevance to the field (scope), accessibility (open access vs. subscription) and time to be cited [36]. The overall impact of Australian research in DFD has been ranked as 10th in the world [23], utilising total citations as an indicator of impact. Globally, the 50 most highly cited publications in DFD range from 231 to 1661 total citations [18], with the highest cited global publication a systematic review on prevention of DFU by Singh et al. [37]. Fifteen of these leading 50 publications globally were review articles, which suggest that reviews may be considered to be a highly credible source of information to researchers in the field. It may also be related to the ease of citing a review rather than an original source, or authors may be limited by journal reference counts, making reviews more favourable to cite. It is therefore not unexpected that the Australian publication with the greatest number of total citations was a Cochrane systematic review by Bergin and Wraight [31] on the effectiveness of silver dressings on management of diabetes‐related foot ulceration, but perhaps it is unexpected that it has a total citation count of one 10th of the most highly cited international DFD paper (*n* = 151). This may be explained by the recency of the substantial increases in DFD research publications in Australia, which suggests that Australia has only recently emerged as a leading country for DFD research in the past decade. However, because of this recency, these outputs have not had equivalent time to be cited like other more established DFD research countries including the United States and the Netherlands. It will be of interest to monitor this over time to determine if Australia sees proportional increases in citations in line with the recent increased output.

The greatest volume of publications by institution was Queensland University of Technology, followed by the University of Sydney and James Cook University. This is likely related to a greater concentration of the most productive authors of Australian DFD research having DFD‐related research programmes in these institutions with some also attracting Australian competitive research funding. Furthermore, James Cook University has also had previous success in gaining >$AU2.6 million of NHMRC Centre of Research Excellence funding for peripheral artery disease [38]. This competitive research funding appears to have been a substantial driver of DFD research productivity in the leading Australian institutions. However, the results of the current study highlight the very low competitive funding sources specifically supporting Australian DFD research overall. A focus on improving this situation in Australia would seem a logical next step to consolidating and driving further increases in Australian DFD research that addresses national and global DFD evidence and burdens. International collaboration was strongest between Australia and the UK, where national healthcare delivery and hospital systems share commonalities. However, the collaboration was also strong with the Netherlands, the United States and New Zealand. This collaborative network is likely related to a rapid parallel increase in Australian authors recently participating in the IWGDF DFD guidelines, where other leading authors are mainly from other leading nations such as the UK, USA and led by the Netherlands. Furthermore, this also likely relates to international authors being attracted or affiliated to Australian institutions, such as Jaap van Netten, the fifth leading Australian affiliated author, who spent a number of years as a research fellow in Australia and maintains an Australian affiliation. Similarly, some Australian DFD researchers have spent time abroad on collaborative visits to establish international collaborations, which also fosters international collaboration, including on programmes such as Churchill or Fulbright fellowships. The Australian international collaborative networks differed from New Zealand DFD researchers, who predominantly worked with Australian, followed by Finland and German researchers [19].

Most of the research publications were published in the Journal of Foot and Ankle Research, which is the official journal of the Australian Podiatry Association and the Royal College of Podiatry in the United Kingdom. Australian researchers who are members of the Australian Podiatry Association are able to publish in the open access, Journal of Foot and Ankle Research at no personal cost, which may be a factor driving submissions. This may also suggest that researchers may rely on this subsidy to publish open access given the lack of reported research funding, reiterating the need for more support to researchers to support publishing open access. Researchers typically aim to publish in leading journals in their field or those with the highest impact factors, or in the highest quartile of journals in the field, which meet the scope of their research, are open access and provide publication in a timely manner [39]. Diabetes Care could be considered a leading journal within the DFD field with an impact factor of 16.2, and was one of the highest 10 journals Australian authors published in with six publications (2%). This journal was similarly placed in the highest 10 publications in the global DFD bibliometric analysis with 125 total publications [23]. Very high impact journals generally seek to publish robust multi‐centre trials, which are more broadly applicable and determine causality. These are currently not widely undertaken in Australian DFD research due to a lack of funding. The Journal of Foot and Ankle Research appears to be more accessible and may also have a greater interest in publishing foot specific DFD research compared to broader diabetes journal with much larger scope. This particular metric will be of interest in future bibliometric analyses as the field matures, as it would be expected that the number of publications in high impact journals will increase.

A previous bibliometric publication reported the characteristics of DFD research according to health dimensions [23], however, to the best of the authors' knowledge, this is the first bibliometric study to categorise research using an established research activity classification system developed by all stakeholders that influence clinical research. The majority of original Australian research pertained to research relating to etiology, existing treatments and health services research. This is understandable considering the limited funding associated with Australian DFD research and evaluating essentially existing patients, treatments and services is likely comparatively less resource intensive than researching on much larger populations need for prevention studies and new treatments in trials. These existing types of research are not without challenges, however, and requires resources to create databases, validate tools and train clinicians, however does lend itself to in kind contributions. It is commendable that Australian researchers are investigating aetiological factors associated with DFD. Some important discoveries have occurred as a result of such research, including identifying that younger‐onset type 2 diabetes is an independent risk factor for poor healing and hospitalisation [40, 41]. However, these important discoveries are based on large prospective multi‐centre cohort studies, which are more representative and powerful than single‐centre retrospective data. It is recommended that more prospective multi‐centre studies would be particularly beneficial and add to the national evidence base in DFD research.

The evaluation of existing treatments was the second most common research category, with a focus on the evaluation of existing medical devices. People with DFD often interact with medical devices, which include footwear, offloading devices, vascular assessment equipment and wound dressings and interventions. Smaller publications investigating these types of existing and often well used medical devices can also be conducted relatively inexpensively in organised clinical settings. However, very few publications in this category implemented randomised controlled trials evaluating a new treatment or intervention. This also appears consistent with the funding data. Randomised controlled trials are very resource intensive, and in Australia are funded primarily through the federal government's nationally competitive category one programmes. Similarly, low numbers of randomised controlled trials have been previously described in other DFD bibliometric analyses [19, 21], which also suggests that there are challenges in executing randomised controlled trials in this area of research.

There has been very little Australian research in DFD prevention with only one percent of total publications focused on prevention. A recent bibliometric analysis of New Zealand‐based DFD publications showed a greater local research focus on screening and prevention (30%) compared with Australia (<1%) [19]. It is currently not possible to draw any further comparisons with other countries as the research categories have not been evaluated. The low numbers of publications in prevention demonstrates that this remains a low priority for researchers working with people with DFD in Australia. This research focus may be due to a number of factors. Most people with active DFD will engage in secondary or hospital care as part of their healthcare, where the research infrastructure (including relationships with universities and research centres) is likely to be more mature than community or primary healthcare. In addition, on a national level, historical research funding shows a preference for research in tertiary‐level settings. Furthermore, to demonstrate effectiveness in prevention studies requires much larger populations than in treatment studies, which obviously also impacts on resources required for prevention studies [42]. This lack of emphasis on prevention is concerning considering DFD is a leading cause of hospitalisation, amputation, poor quality of life [43] and imposes a huge disease burden on an individual and society [3], and it has been estimated that effective prevention of the development of DFD can significantly reduce these disease burdens [44]. More research is needed to inform clinicians and policy makers how we can effectively prevent DFD in Australians.

Funding categorisation showed that the Australian Government awarded very little nationally competitive funding to DFD, with 7% of all Australian DFD research over the entire period funded by external nationally competitive sources. Overall general research funding in Australia is lower than the Organisation for Economic Cooperation and Development average with Australia ranking 10th behind countries including Korea, Sweden, the United States and Japan [45]. The NHMRC reported a decrease in the total funding pool attributed to diabetes‐related research in recent times from $65 million in 2013, to $42 million in 2021, with similar decreases seen in other chronic conditions including cardiovascular disease and obesity [46]. This decrease in funding is despite the increasing prevalence of diabetes and its associated complications in the Australian population [47], and despite a strategy to support Australian DFD research output published by Diabetes Feet Australia, which included recommendations for the establishment of national research priorities, development of a clinical trials network and proportional investment in DFD research [13]. Success rates for NHMRC researcher support (fellowship) schemes and project grants have also been negatively impacted and were higher in 2013 than in 2021 [48]. The current reported success rates are much lower than similar national schemes in the United Kingdom and the United States [49, 50]. Furthermore, it was highlighted that NHMRC funding does not completely align with societal burden of disease and some national health priorities are better funded than others. However, diabetes was reported to have a higher proportion of NHMRC funding compared to other priority health areas [51]. It is currently unclear whether researchers in the field of DFD apply for competitive funding or what the specific success rates are. Overall, the low apparent funding rate not only underscores the resilience of Australian researchers in DFD to complete their research without significant funding, but is probably also contributing to the lack of more resource intensive clinical trials in this area.

Some limitations of the current study should be considered. Research funding reporting may be under‐reported and therefore may not adequately represent actual sources of funding. Peer reviewed publications that are not indexed in Scopus ^®^ would not have appeared in our search and therefore would not have been included, so some publications may have been missed. Additionally, our search terms may not have captured all of the research conducted in the field. However, this was based on previous bibliometric publications in the field to reduce the likelihood of such an occurrence and improve our comparability to those studies [19, 21]. Our exclusion criteria included letters and short reports which may have excluded some Australian original research publications, which are published in high‐impact journals and/or are highly cited [4, 41, 52]. The exclusion of guidelines also would have influenced the median citations of publications, as guidelines are commonly cited and some include Australian authors [6, 53]. Further, whilst we used two independent authors to perform the abstract and full text screening process, the categorisation of research and funding classification was completed by a single author, with only a random sample checked by a second author, which may have led to some inaccuracies.

## 5 CONCLUSION

Whilst the volume of scholarly work of Australian DFD researchers has dramatically increased in the last decade, there remains very small amounts of nationally competitive funding attributed to the field. Thus, many Australian DFD researchers are conducting research with little to no funding, and research outputs are based on feasible and pragmatic studies which are not necessarily being driven by need or priorities. Whilst this suggests that Australian DFD researchers are resilient and able to produce high volumes of output despite a lack of funding, there is a level of risk to the future viability of the field without substantial new competitive funding. In particular, future research should consider greater focus on prevention studies and the development and trials of new treatments, as the volume of local research in this area was substantially lacking.

## AUTHOR CONTRIBUTIONS

Peta E. Tehan and Matthew R. Carroll contributed to the conception, design, search, eligibility assessments, data extraction, analyses and interpretation, and wrote the paper. Byron M. Perrin, Peter A. Lazzarini, Ibrahim S. Al‐Busaidi contributed to the design, interpreted the data and critically reviewed the paper. Byron M. Perrin contributed to categorising research activity types and Peter A. Lazzarini to categorising research funding. All authors approved the final paper for submission.

## CONFLICT OF INTEREST STATEMENT

None of the authors have any conflicts or competing interests to declare. BMP chair and PAL past chair of Diabetes Feet Australia. PT, PAL and MC are on the editorial board of the Journal of Foot and Ankle Research.

## ETHICS STATEMENT

Not applicable.

## CONSENT FOR PUBLICATION

Not applicable.

## Supporting information

Supporting Information S1

## Data Availability

All data collected for the study is presented in the body of the manuscript or in the supplementary files.
