# Supplementary material for: How far has diabetes‐related foot disease research progressed in Australia? A bibliometric review (1970–2023)
Source: J Foot Ankle Res. 2024 Apr 16;17(2):e12012. doi: 10.1002/jfa2.12012 (PMC11080706; doi:10.1002/jfa2.12012)
Supplement: Supplementary file 1 — Supporting Information S1 [file JFA2-17-e12012-s001.docx]

**Appendix 1: List of included publications**

| Authors | Titles | Year | Source title | Volume | Issue | DOI |  | |
| --- | --- | --- | --- | --- | --- | --- | --- | --- |
| Ababneh A.; Edwards H.; Lazzarini P.; Finlayson K. | A qualitative exploration of adherence to wearing removable cast walkers in patients with diabetic foot ulcers | 2023 | Journal of Wound Care | 32 | 7 | 10.12968/jowc.2023.32.7.456 | |  |
| Ababneh A.; Finlayson K.; Edwards H.; Armstrong D.G.; Najafi B.; van Netten J.J.; Lazzarini P.A. | The Validity and Reliability of Self-Reported Adherence to Using Offloading Treatment in People with Diabetes-Related Foot Ulcers | 2023 | Sensors | 23 | 9 | 10.3390/s23094423 | |  |
| Ababneh A.; Finlayson K.; Edwards H.; Lazzarini P.A. | Factors associated with adherence to using removable cast walker treatment among patients with diabetes-related foot ulcers | 2022 | BMJ Open Diabetes Research and Care | 10 | 1 | 10.1136/bmjdrc-2021-002640 | |  |
| Ababneh A.; Finlayson K.; Lazzarini P.; Edwards H. | Cross-Cultural Adaptation and Reliability Testing of Arabic Versions of Several Diabetic Foot Psychosocial Scales | 2023 | International Journal of Lower Extremity Wounds | 22 | 2 | 10.1177/15347346211016699 | |  |
| Abu-Qamar M.Z.; Kemp V.; Whitehead L. | The reported external traumas among people with diabetes-related foot ulcers and their outcomes: A systematic review of case reports | 2022 | International Wound Journal | 19 | 6 | 10.1111/iwj.13731 | |  |
| Adam D.J.; Raptis S.; Fitridge R.A. | Trends in the presentation and surgical management of the acute diabetic foot | 2006 | European Journal of Vascular and Endovascular Surgery | 31 | 2 | 10.1016/j.ejvs.2005.05.039 | |  |
| Ahmed M.U.; Tannous W.K.; Agho K.E.; Henshaw F.; Turner D.; Simmons D. | Social determinants of diabetes-related foot disease among older adults in New South Wales, Australia: evidence from a population-based study | 2021 | Journal of Foot and Ankle Research | 14 | 1 | 10.1186/s13047-021-00501-8 | |  |
| Ahmed M.U.; Tannous W.K.; Agho K.E.; Henshaw F.; Turner D.; Simmons D. | Prevalence and burden of diabetes-related foot disease in new south wales, australia: Evidence from the 45 and up study survey data linked with health services data | 2021 | International Journal of Environmental Research and Public Health | 18 | 21 | 10.3390/ijerph182111528 | |  |
| Ahmed M.U.; Tannous W.K.; Agho K.E.; Henshaw F.; Turner D.; Simmons D. | The burden of diabetes-related foot disease among older adults in Australia | 2022 | International Wound Journal | 19 | 7 | 10.1111/iwj.13781 | |  |
| Aitken E.; Hiew J.; Hamilton E.J.; Manning L.; Ritter J.C.; Raby E.; Gittings P.M. | Exercise in adults admitted to hospital with diabetes-related foot ulcers: a pilot study of feasibility and safety | 2023 | Journal of Foot and Ankle Research | 16 | 1 | 10.1186/s13047-023-00616-0 | |  |
| Alahakoon C.; Fernando M.; Galappaththy C.; Lazzarini P.; Moxon J.V.; Jones R.; Golledge J. | Repeatability, Completion Time, and Predictive Ability of Four Diabetes-Related Foot Ulcer Classification Systems | 2023 | Journal of Diabetes Science and Technology | 17 | 1 | 10.1177/1932296820986548 | |  |
| Alahakoon C.; Fernando M.; Galappaththy C.; Matthews E.O.; Lazzarini P.; Moxon J.V.; Golledge J. | Meta-analyses of randomized controlled trials reporting the effect of home foot temperature monitoring, patient education or offloading footwear on the incidence of diabetes-related foot ulcers | 2020 | Diabetic Medicine | 37 | 8 | 10.1111/dme.14323 | |  |
| Alahakoon C.; Singh T.P.; Galappaththy C.; Charles J.; Fernando M.; Lazzarini P.; Moxon J.V.; Golledge J. | Risk Factors for Hospital Re-admission for Diabetes Related Foot Disease: A Prospective Cohort Study | 2023 | European Journal of Vascular and Endovascular Surgery | 66 | 2 | 10.1016/j.ejvs.2023.05.016 | |  |
| Alahakoon C.; Singh T.P.; Morris D.; Charles J.; Fernando M.; Lazzarini P.; Moxon J.V.; Golledge J. | Cohort Study Examining the Presentation, Distribution, and Outcomes of Peripheral Artery Disease in Aboriginal, Torres Strait Islander, and Non-Indigenous Australians | 2023 | European Journal of Vascular and Endovascular Surgery | 66 | 2 | 10.1016/j.ejvs.2023.05.027 | |  |
| Alahakoon C.; Thanigaimani S.; Seng L.; Fernando M.; Lazzarini P.; Golledge J. | Editor's Choice – A Systematic Review and Meta-Analysis of the Incidence and Risk Factors for Re-admission to Hospital in People with Diabetes Related Foot Disease | 2023 | European Journal of Vascular and Endovascular Surgery | 66 | 2 | 10.1016/j.ejvs.2023.05.007 | |  |
| Aliahmad B.; Tint A.N.; Poosapadi Arjunan S.; Rani P.; Kumar D.K.; Miller J.; Zajac J.D.; Wang G.; Ekinci E.I. | Is Thermal Imaging a Useful Predictor of the Healing Status of Diabetes-Related Foot Ulcers? A Pilot Study | 2019 | Journal of Diabetes Science and Technology | 13 | 3 | 10.1177/1932296818803115 | |  |
| Alzubaidi L.; Fadhel M.A.; Oleiwi S.R.; Al-Shamma O.; Zhang J. | DFU_QUTNet: diabetic foot ulcer classification using novel deep convolutional neural network | 2020 | Multimedia Tools and Applications | 79 | 21-22 | 10.1007/s11042-019-07820-w | |  |
| Argarini R.; McLaughlin R.A.; Joseph S.Z.; Naylor L.H.; Carter H.H.; Yeap B.B.; Jansen S.J.; Green D.J. | Optical coherence tomography: A novel imaging approach to visualize and quantify cutaneous microvascular structure and function in patients with diabetes | 2020 | BMJ Open Diabetes Research and Care | 8 | 1 | 10.1136/bmjdrc-2020-001479 | |  |
| Au-Yeung K.L.; Selvaraj C.; Amin T.; K Ma L.; Bennett M.H. | Effect of enriched oxygen inhalation on lower limb skin temperatures in diabetic and healthy humans: a pilot study | 2022 | Diving and Hyperbaric Medicine | 52 | 1 | 10.28920/dhm52.1.2-6 | |  |
| Baba M.; Davis W.A.; Davis T.M.E. | A longitudinal study of foot ulceration and its risk factors in community-based patients with type 2 diabetes: The Fremantle Diabetes Study | 2014 | Diabetes Research and Clinical Practice | 106 | 1 | 10.1016/j.diabres.2014.07.021 | |  |
| Baba M.; Davis W.A.; Norman P.E.; Davis T.M.E. | Temporal changes in the prevalence and associates of foot ulceration in type 2 diabetes: The Fremantle Diabetes Study | 2015 | Journal of Diabetes and its Complications | 29 | 3 | 10.1016/j.jdiacomp.2015.01.008 | |  |
| Baba M.; Davis W.A.; Norman P.E.; Davis T.M.E. | Temporal changes in the prevalence and associates of diabetes-related lower extremity amputations in patients with type 2 diabetes: The Fremantle Diabetes Study | 2015 | Cardiovascular Diabetology | 14 | 1 | 10.1186/s12933-015-0315-z | |  |
| Baba M.; Foley L.; Davis W.A.; Davis T.M.E. | Self-awareness of foot health status in patients with type 2 diabetes: The Fremantle Diabetes Study Phase II | 2014 | Diabetic Medicine | 31 | 11 | 10.1111/dme.12521 | |  |
| Barakat-Johnson M.; Kita B.; Jones A.; Burger M.; Airey D.; Stephenson J.; Leong T.; Pinkova J.; Frank G.; Ko N.; Kirk A.; Frotjold A.; White K.; Coyer F. | The viability and acceptability of a Virtual Wound Care Command Centre in Australia | 2022 | International Wound Journal | 19 | 7 | 10.1111/iwj.13782 | |  |
| Barber C.; Watt A.; Pham C.; Humphreys K.; Penington A.; Mutimer K.; Edwards M.; Maddern G. | Influence of bioengineered skin substitutes on diabetic foot ulcer and venous leg ulcer outcomes. | 2008 | Journal of wound care | 17 | 12 | 10.12968/jowc.2008.17.12.31766 | |  |
| Barth R.; Campbell L.V.; Allen S.; Jupp J.J.; Chisholm D.J. | Intensive Education Improves Knowledge, Compliance, and Foot Problems in Type 2 Diabetes | 1991 | Diabetic Medicine | 8 | 2 | 10.1111/j.1464-5491.1991.tb01555.x | |  |
| Barwick A.; Tessier J.; Mirow J.; de Jonge X.J.; Chuter V. | Computed tomography derived bone density measurement in the diabetic foot | 2017 | Journal of Foot and Ankle Research | 10 | 1 | 10.1186/s13047-017-0192-7 | |  |
| Barwick A.L.; de Jonge X.A.K.J.; Tessier J.W.; Ho A.; Chuter V.H. | The effect of diabetic neuropathy on foot bones: A systematic review and meta-analysis | 2014 | Diabetic Medicine | 31 | 2 | 10.1111/dme.12347 | |  |
| Barwick A.L.; Hurn S.E.; van Netten J.J.; Reed L.F.; Lazzarini P.A. | Factors associated with wearing inadequate outdoor footwear in populations at risk of foot ulceration: A cross-sectional study | 2019 | PLoS ONE | 14 | 2 | 10.1371/journal.pone.0211140 | |  |
| Barwick A.L.; Tessier J.W.; De Jonge X.J.; Ivers J.R.; Chuter V.H. | Peripheral sensory neuropathy is associated with altered postocclusive reactive hyperemia in the diabetic foot | 2016 | BMJ Open Diabetes Research and Care | 4 | 1 | 10.1136/bmjdrc-2016-000235 | |  |
| Barwick A.L.; Tessier J.W.; Janse de Jonge X.; Chuter V.H. | Foot bone density in diabetes may be unaffected by the presence of neuropathy | 2016 | Journal of Diabetes and its Complications | 30 | 6 | 10.1016/j.jdiacomp.2016.04.013 | |  |
| Bechara N.; Gunton J.E.; Flood V.; Hng T.-M.; McGloin C. | Associations between nutrients and foot ulceration in diabetes: A systematic review | 2021 | Nutrients | 13 | 8 | 10.3390/nu13082576 | |  |
| Begg L.; McLaughlin P.; Vicaretti M.; Fletcher J.; Burns J. | Total contact cast wall load in patients with a plantar forefoot ulcer and diabetes | 2016 | Journal of Foot and Ankle Research | 9 | 1 | 10.1186/s13047-015-0119-0 | |  |
| Behrendt C.-A.; Sigvant B.; Kuchenbecker J.; Grima M.J.; Schermerhorn M.; Thomson I.A.; Altreuther M.; Setacci C.; Svetlikov A.; Laxdal E.H.; Goncalves F.B.; Secemsky E.A.; Debus E.S.; Cassar K.; Beiles B.; Beck A.W.; Mani K.; Bertges D. | Editor's Choice – International Variations and Sex Disparities in the Treatment of Peripheral Arterial Occlusive Disease: A Report from VASCUNET and the International Consortium of Vascular Registries | 2020 | European Journal of Vascular and Endovascular Surgery | 60 | 6 | 10.1016/j.ejvs.2020.08.027 | |  |
| Behrendt C.-A.; Sigvant B.; Szeberin Z.; Beiles B.; Eldrup N.; Thomson I.A.; Venermo M.; Altreuther M.; Menyhei G.; Nordanstig J.; Clarke M.; Rieß H.C.; Björck M.; Debus E.S. | International Variations in Amputation Practice: A VASCUNET Report | 2018 | European Journal of Vascular and Endovascular Surgery | 56 | 3 | 10.1016/j.ejvs.2018.04.017 | |  |
| Ben chmo M.; Matricciani L.; Kumar S.; Graham K. | “I was trying to look after myself, but I really wasn’t”: Understanding patient’s perspectives on risk factors for lower extremity amputations | 2022 | Journal of Foot and Ankle Research | 15 | 1 | 10.1186/s13047-022-00589-6 | |  |
| Bennett P.J.; Stocks A.E.; Whittam D.J. | Analysis of risk factors for neuropathic foot ulceration in diabetes mellitus | 1996 | Journal of the American Podiatric Medical Association | 86 | 3 | 10.7547/87507315-86-3-112 | |  |
| Bergin S.; Naidoo P.; Williams C.M. | A radiological severity scale to measure the impact of Charcot's Neuroarthropathy: An observational study | 2020 | Journal of Foot and Ankle Research | 13 | 1 | 10.1186/s13047-020-0375-5 | |  |
| Bergin S.M.; Brand C.A.; Colman P.G.; Campbell D.A. | A questionnaire for determining prevalence of diabetes related foot disease (Q-DFD): Construction and validation | 2009 | Journal of Foot and Ankle Research | 2 | 1 | 10.1186/1757-1146-2-34 | |  |
| Bergin S.M.; Brand C.A.; Colman P.G.; Campbell D.A. | The impact of socio-economic disadvantage on rates of hospital separations for diabetes-related foot disease in Victoria, Australia | 2011 | Journal of Foot and Ankle Research | 4 | 1 | 10.1186/1757-1146-4-17 | |  |
| Bergin S.M.; Wraight P. | Silver based wound dressings and topical agents for treating diabetic foot ulcers. | 2006 | Cochrane database of systematic reviews (Online) | | 1 |  |  | |
| Berhane T.; Jeyaraman K.; Hamilton M.; Falhammar H. | Pressure relieving interventions for the management of diabetes-related foot ulcers: a study from the Northern Territory of Australia | 2022 | ANZ Journal of Surgery | 92 | 4 | 10.1111/ans.17431 | |  |
| Bhamidipaty V.; Dean A.; Yap S.L.; Firth J.; Barron M.; Allard B.; Chan S.T.F. | Second toe systolic pressure measurements are valid substitutes for first toe systolic pressure measurements in diabetic patients: A prospective study | 2015 | European Journal of Vascular and Endovascular Surgery | 49 | 1 | 10.1016/j.ejvs.2014.09.011 | |  |
| Bhatt U.K.; Foo H.Y.; McEvoy M.P.; Tomlinson S.J.; Westphal C.; Harrison J.C.; Oshin O.; Carter S.L. | Is TCC-EZ a Suitable Alternative to Gold Standard Total-Contact Casting? A Plantar Pressure Analysis | 2021 | Journal of the American Podiatric Medical Association | 111 | 5 | 10.7547/8750-7315-111-5.ARTICLE_1 | |  |
| Blatchford L.; Morey P.; McConigley R. | Identifying type 2 diabetes risk classification systems and recommendations for review of podiatric care in an Australian Aboriginal health clinic | 2015 | Journal of Foot and Ankle Research | 8 | 1 | 10.1186/s13047-015-0089-2 | |  |
| Boland-Freitas R.; Ng K. | Assessment of small sensory fiber function in myotonic dystrophy type 1 | 2019 | Muscle and Nerve | 60 | 5 | 10.1002/mus.26673 | |  |
| Bonanno D.R.; Gillies E.J. | Flexor Tenotomy Improves Healing and Prevention of Diabetes-Related Toe Ulcers: A Systematic Review | 2017 | Journal of Foot and Ankle Surgery | 56 | 3 | 10.1053/j.jfas.2017.02.011 | |  |
| Bongaerts B.W.C.; Ziegler D.; Shaw J.E.; Heier M.; Kowall B.; Herder C.; Roden M.; Peters A.; Meisinger C.; Rathmann W. | A clinical screening score for diabetic polyneuropathy: KORA F4 and AusDiab Studies | 2015 | Journal of Diabetes and its Complications | 29 | 1 | 10.1016/j.jdiacomp.2014.09.014 | |  |
| Bower V.M.; Hobbs M. | Validation of the basic foot screening checklist: A population screening toot for identifying foot ulcer risk in people with diabetes mellitus | 2009 | Journal of the American Podiatric Medical Association | 99 | 4 | 10.7547/0980339 | |  |
| Brookes J.D.L.; Jaya J.S.; Tran H.; Vaska A.; Werner-Gibbings K.; D’Mello A.C.; Wong J.; Lemoh C.N.; Saunder A.C.; Yii M.K. | Broad-Ranging Nutritional Deficiencies Predict Amputation in Diabetic Foot Ulcers | 2020 | International Journal of Lower Extremity Wounds | 19 | 1 | 10.1177/1534734619876779 | |  |
| Burns J.; Begg L. | Optimizing the offloading properties of the total contact cast for plantar foot ulceration | 2011 | Diabetic Medicine | 28 | 2 | 10.1111/j.1464-5491.2010.03135.x | |  |
| Burns J.; Wegener C.; Begg L.; Vicaretti M.; Fletcher J. | Randomized trial of custom orthoses and footwear on foot pain and plantar pressure in diabetic peripheral arterial disease | 2009 | Diabetic Medicine | 26 | 9 | 10.1111/j.1464-5491.2009.02799.x | |  |
| Causby R.S.; Pod M.; Jones S. | Dressing plantar wounds with foam dressings, is it too much pressure? | 2011 | Diabetic Foot and Ankle | 2 |  |  |  | |
| Chan K.H.; O'Connell R.L.; Sullivan D.R.; Hoffmann L.S.; Rajamani K.; Whiting M.; Donoghoe M.W.; Vanhala M.; Hamer A.; Yu B.; Stocker R.; Ng M.K.C.; Keech A.C. | Plasma total bilirubin levels predict amputation events in type 2 diabetes mellitus: The Fenofibrate Intervention and Event Lowering in Diabetes (FIELD) study | 2013 | Diabetologia | 56 | 4 | 10.1007/s00125-012-2818-4 | |  |
| Chen P.; Callisaya M.; Wills K.; Greenaway T.; Winzenberg T. | Associations of health literacy with risk factors for diabetic foot disease: A cross-sectional analysis of the Southern Tasmanian Health Literacy and Foot Ulcer Development in Diabetes Mellitus Study | 2019 | BMJ Open | 9 | 7 | 10.1136/bmjopen-2018-025349 | |  |
| Chen P.; Callisaya M.; Wills K.; Greenaway T.; Winzenberg T. | Health Literacy predicts incident foot ulcers after 4 years – the SHELLED cohort study | 2023 | Journal of Foot and Ankle Research | 16 | 1 | 10.1186/s13047-023-00644-w | |  |
| Chen P.Y.; Elmer S.; Callisaya M.; Wills K.; Greenaway T.M.; Winzenberg T.M. | Associations of health literacy with diabetic foot outcomes: a systematic review and meta-analysis | 2018 | Diabetic Medicine | 35 | 11 | 10.1111/dme.13694 | |  |
| Cheng Q.; Graves N.; Pacella R.E. | Economic Evaluations of Guideline-Based Care for Chronic Wounds: a Systematic Review | 2018 | Applied Health Economics and Health Policy | 16 | 5 | 10.1007/s40258-018-0403-9 | |  |
| Cheng Q.; Lazzarini P.A.; Gibb M.; Derhy P.H.; Kinnear E.M.; Burn E.; Graves N.; Norman R.E. | A cost-effectiveness analysis of optimal care for diabetic foot ulcers in Australia | 2017 | International Wound Journal | 14 | 4 | 10.1111/iwj.12653 | |  |
| Chuter V.; Payne C. | Limited joint mobility and plantar fascia function in Charcot's neuroarthropathy | 2001 | Diabetic Medicine | 18 | 7 | 10.1046/j.1464-5491.2001.00527.x | |  |
| Chuter V.; West M.; Hawke F.; Searle A. | Where do we stand? the availability and efficacy of diabetes related foot health programs for Aboriginal and Torres Strait Islander Australians: A systematic review | 2019 | Journal of Foot and Ankle Research | 12 | 1 | 10.1186/s13047-019-0326-1 | |  |
| Chuter V.H.; Spink M.J.; David M.; Lanting S.; Searle A. | Clinical foot measurements as a proxy for plantar pressure testing in people with diabetes | 2021 | Journal of Foot and Ankle Research | 14 | 1 | 10.1186/s13047-021-00494-4 | |  |
| Colagiuri S.; Marsden L.L.; Naidu V.; Taylor L. | The use of orthotic devices to correct plantar callus in people with diabetes | 1995 | Diabetes Research and Clinical Practice | 28 | 1 | 10.1016/0168-8227(95)01050-N | |  |
| Collins R.; Burrows T.; Donnelly H.; Tehan P.E. | Macronutrient and micronutrient intake of individuals with diabetic foot ulceration: A short report | 2022 | Journal of Human Nutrition and Dietetics | 35 | 5 | 10.1111/jhn.12974 | |  |
| Commons R.J.; Raby E.; Athan E.; Bhally H.; Chen S.; Guy S.; Ingram P.R.; Lai K.; Lemoh C.; Lim L.-L.; Manning L.; Miyakis S.; O'Reilly M.; Roberts A.; Sehu M.; Torda A.; Vicaretti M.; Lazzarini P.A. | Managing diabetic foot infections: A survey of Australasian infectious diseases clinicians | 2018 | Journal of Foot and Ankle Research | 11 | 1 | 10.1186/s13047-018-0256-3 | |  |
| Commons R.J.; Robinson C.H.; Gawler D.; Davis J.S.; Price R.N. | High burden of diabetic foot infections in the top end of Australia: An emerging health crisis (DEFINE study) | 2015 | Diabetes Research and Clinical Practice | 110 | 2 | 10.1016/j.diabres.2015.09.016 | |  |
| Corbett C.; Jolley J.; Barson E.; Wraight P.; Perrin B.; Fisher C. | Cognition and Understanding of Neuropathy of Inpatients Admitted to a Specialized Tertiary Diabetic Foot Unit With Diabetes-Related Foot Ulcers | 2019 | International Journal of Lower Extremity Wounds | 18 | 3 | 10.1177/1534734619862085 | |  |
| Coyle M.E.; Francis K.; Chapman Y. | Self-management activities in diabetes care: A systematic review | 2013 | Australian Health Review | 37 | 4 | 10.1071/AH13060 | |  |
| Crowley B.; Drovandi A.; Seng L.; Fernando M.E.; Ross D.; Golledge J. | Patient Perspectives on the Burden and Prevention of Diabetes-Related Foot Disease | 2023 | Science of Diabetes Self-Management and Care | 49 | 3 | 10.1177/26350106231170531 | |  |
| Dallimore S.M.; Kaminski M.R. | Tendon lengthening and fascia release for healing and preventing diabetic foot ulcers: A systematic review and meta-analysis | 2015 | Journal of Foot and Ankle Research | 8 | 1 | 10.1186/s13047-015-0085-6 | |  |
| Dallimore S.M.; Puli N.; Kim D.; Kaminski M.R. | Infrared dermal thermometry is highly reliable in the assessment of patients with Charcot neuroarthropathy | 2020 | Journal of Foot and Ankle Research | 13 | 1 | 10.1186/s13047-020-00421-z | |  |
| Dando C.; Lane G.; Bowen C.; Henshaw F. | The evaluation of podiatrists, with knowledge and training in diagnostic musculoskeletal ultrasound, to describe sonographic images of diabetic foot wounds in the United Kingdom and Australia | 2022 | Journal of Foot and Ankle Research | 15 | 1 | 10.1186/s13047-022-00511-0 | |  |
| Davis W.A.; Norman P.E.; Bruce D.G.; Davis T.M. | Predictors, consequences and costs of diabetes-related lower extremity amputation complicating type 2 diabetes: the Fremantle Diabetes Study. | 2006 | Diabetologia | 49 | 11 | 10.1007/s00125-006-0431-0 | |  |
| Dehghani C.; Russell A.W.; Perkins B.A.; Malik R.A.; Pritchard N.; Edwards K.; Shahidi A.M.; Srinivasan S.; Efron N. | A rapid decline in corneal small fibers and occurrence of foot ulceration and Charcot foot | 2016 | Journal of Diabetes and its Complications | 30 | 8 | 10.1016/j.jdiacomp.2016.07.004 | |  |
| Delbridge L.; Appleberg M.; Reeve T.S. | Factors associated with development of foot lesions in the diabetic | 1983 | Surgery | 93 | 1 PART 1 |  |  | |
| Delbridge L.; Perry P.; Marr S.; Arnold N.; Yue D.K.; Turtle J.R.; Reeve T.S. | Limited Joint Mobility in the Diabetic Foot: Relationship to Neuropathic Ulceration | 1988 | Diabetic Medicine | 5 | 4 | 10.1111/j.1464-5491.1988.tb01000.x | |  |
| Diab J.; O'Hara J.; Pye M.; Parker C.; Maitz P.K.M.; Issler-Fisher A. | Foot burns: A comparative analysis of diabetic and non-diabetic patients | 2021 | Burns | 47 | 3 | 10.1016/j.burns.2020.07.024 | |  |
| Diacogiorgis D.; Perrin B.M.; Kingsley M.I.C. | Factors impacting the evidence-based assessment, diagnosis and management of Acute Charcot Neuroarthropathy: a systematic review | 2021 | Journal of Foot and Ankle Research | 14 | 1 | 10.1186/s13047-021-00469-5 | |  |
| Dillon M.P.; Fortington L.V.; Akram M.; Erbas B.; Kohler F. | Geographic variation of the incidence rate of lower limb amputation in Australia from 2007-12 | 2017 | PLoS ONE | 12 | 1 | 10.1371/journal.pone.0170705 | |  |
| Dillon M.P.; Quigley M.; Fatone S. | A systematic review describing incidence rate and prevalence of dysvascular partial foot amputation; how both have changed over time and compare to transtibial amputation | 2017 | Systematic Reviews | 6 | 1 | 10.1186/s13643-017-0626-0 | |  |
| Dinh N.T.T.; De Graaff B.; Campbell J.A.; Jose M.D.; Burgess J.; Saunder T.; Kitsos A.; Otahal P.; Palmer A.J. | Risk of hospital admission or emergency department presentation due to diabetes complications: a retrospective cohort study in Tasmania, Australia | 2023 | Australian Health Review | 47 | 3 | 10.1071/AH22271 | |  |
| Dinh N.T.T.; de Graaff B.; Campbell J.A.; Jose M.D.; John B.; Saunder T.; Kitsos A.; Wiggins N.; Palmer A.J. | Costs of major complications in people with and without diabetes in Tasmania, Australia | 2022 | Australian health review : a publication of the Australian Hospital Association | 46 | 6 | 10.1071/AH22180 | |  |
| Donaghue K.C.; Bonney M.; Simpson J.M.; Schwingshandl J.; Fung A.T.W.; Howard N.J.; Silinka M. | Autonomic and Peripheral Nerve Function in Adolescents With and Without Diabetes | 1993 | Diabetic Medicine | 10 | 7 | 10.1111/j.1464-5491.1993.tb00142.x | |  |
| Donaghue K.C.; Fung A.T.W.; Fairchild J.M.; Howard N.J.; Silink M. | Prospective assessment of autonomic and peripheral nerve function in adolescents with diabetes | 1996 | Diabetic Medicine | 13 | 1 | 10.1002/(SICI)1096-9136(199601)13:1<65::AID-DIA997>3.0.CO;2-W | |  |
| Donnelly H.R.; Collins C.E.; Haslam R.; White D.; Tehan P.E. | Perceptions of Diet Quality, Advice, and Dietary Interventions in Individuals with Diabetes-Related Foot Ulceration; A Qualitative Research Study | 2022 | Nutrients | 14 | 12 | 10.3390/nu14122457 | |  |
| Drovandi A.; Seng L.; Crowley B.; Fernando M.E.; Evans R.; Golledge J. | Health Professionals’ Opinions About Secondary Prevention of Diabetes-Related Foot Disease | 2022 | Science of Diabetes Self-Management and Care | 48 | 5 | 10.1177/26350106221112115 | |  |
| Drovandi A.; Wong S.; Seng L.; Crowley B.; Alahakoon C.; Banwait J.; Fernando M.E.; Golledge J. | Remotely Delivered Monitoring and Management of Diabetes-Related Foot Disease: An Overview of Systematic Reviews | 2023 | Journal of Diabetes Science and Technology | 17 | 1 | 10.1177/19322968211012456 | |  |
| Duffin A. | New approach to reducing high plantar pressures in adolescents with diabetes | 1999 | Clinical Biomechanics | 14 | 8 |  |  | |
| Duffin A.C.; Lam A.; Kidd R.; Chan A.K.F.; Donaghue K.C. | Ultrasonography of plantar soft tissues thickness in young people with diabetes | 2002 | Diabetic Medicine | 19 | 12 | 10.1046/j.1464-5491.2002.00850.x | |  |
| Esparza J.; Gudimetla P.; De Silva S.; Unsworth C.A. | An early warning system for diabetic automobile drivers with peripheral neuropathy | 2021 | Disability and Rehabilitation: Assistive Technology | 16 | 6 | 10.1080/17483107.2019.1686076 | |  |
| Ewald D.; Patel M.; Hall G. | Hospital separations indicate increasing need for prevention of diabetic foot complications in central Australia | 2001 | The Australian journal of rural health | 9 | 6 | 10.1046/j.1038-5282.2001.00371.x | |  |
| Faris I.; Duncan H. | Skin perfusion pressure in the prediction of healing in diabetic patients with ulcers or gangrene of the foot | 1985 | Journal of Vascular Surgery | 2 | 4 | 10.1016/0741-5214(85)90005-9 | |  |
| Faris I.; Duncan H.; Young C. | Factors affecting outcome of diabetic patients with foot ulcers or gangrene | 1988 | Journal of Cardiovascular Surgery | 29 | 6 |  |  | |
| Fazekas-Lavu M.; Tonks K.T.T.; Samaras K. | Benchmarks of diabetes care in men living with treated HIV-infection: A tertiary center experience | 2018 | Frontiers in Endocrinology | 9 | OCT | 10.3389/fendo.2018.00634 | |  |
| Featherston J.; Wijlens A.M.; van Netten J.J. | Is a Left-to-Right >2.2°C Difference a Valid Measurement to Predict Diabetic Foot Ulceration in People with Diabetes and a History of Diabetic Foot Ulceration? | 2021 | International Journal of Lower Extremity Wounds | | | 10.1177/15347346211062719 | |  |
| Fernando M.; Crowther R.; Lazzarini P.; Sangla K.; Cunningham M.; Buttner P.; Golledge J. | Biomechanical characteristics of peripheral diabetic neuropathy: A systematic review and meta-analysis of findings from the gait cycle, muscle activity and dynamic barefoot plantar pressure | 2013 | Clinical Biomechanics | 28 | 8 | 10.1016/j.clinbiomech.2013.08.004 | |  |
| Fernando M.; Crowther R.G.; Cunningham M.; Lazzarini P.A.; Sangla K.S.; Buttner P.; Golledge J. | The reproducibility of acquiring three dimensional gait and plantar pressure data using established protocols in participants with and without type 2 diabetes and foot ulcers | 2016 | Journal of Foot and Ankle Research | 9 | 1 | 10.1186/s13047-016-0135-8 | |  |
| Fernando M.E.; Blanchette V.; Mishra R.; Zulbaran-Rojas A.; Rowe V.; Mills J.L.; Armstrong D.G.; Najafi B. | Frailty in People with Chronic Limb Threatening Ischemia and Diabetes-Related Foot Ulcers: A Systematic Review | 2023 | Annals of Vascular Surgery | 89 |  | 10.1016/j.avsg.2022.09.057 | |  |
| Fernando M.E.; Crowther R.G.; Lazzarini P.A.; Sangla K.S.; Buttner P.; Golledge J. | Gait parameters of people with diabetes-related neuropathic plantar foot ulcers | 2016 | Clinical Biomechanics | 37 |  | 10.1016/j.clinbiomech.2016.06.006 | |  |
| Fernando M.E.; Crowther R.G.; Lazzarini P.A.; Sangla K.S.; Wearing S.; Buttner P.; Golledge J. | Plantar pressures are higher in cases with diabetic foot ulcers compared to controls despite a longer stance phase duration | 2016 | BMC Endocrine Disorders | 16 | 1 | 10.1186/s12902-016-0131-9 | |  |
| Fernando M.E.; Crowther R.G.; Lazzarini P.A.; Sangla K.S.; Wearing S.; Buttner P.; Golledge J. | Within- and Between-Body-Site Agreement of Skin Autofluorescence Measurements in People With and Without Diabetes-Related Foot Disease | 2019 | Journal of Diabetes Science and Technology | 13 | 5 | 10.1177/1932296819853555 | |  |
| Fernando M.E.; Crowther R.G.; Lazzarini P.A.; Sangla K.S.; Wearing S.; Buttner P.; Golledge J. | Gait in people with nonhealing diabetes-related plantar ulcers | 2019 | Physical Therapy | 99 | 12 | 10.1093/ptj/pzz119 | |  |
| Fernando M.E.; Crowther R.G.; Lazzarini P.A.; Yogakanthi S.; Sangla K.S.; Buttner P.; Jones R.; Golledge J. | Plantar pressures are elevated in people with longstanding diabetes-related foot ulcers during follow-up | 2017 | PLoS ONE | 12 | 8 | 10.1371/journal.pone.0181916 | |  |
| Fernando M.E.; Crowther R.G.; Pappas E.; Lazzarini P.A.; Cunningham M.; Sangla K.S.; Buttner P.; Golledge J. | Plantar pressure in diabetic peripheral neuropathy patients with active foot ulceration, previous ulceration and no history of ulceration: A meta-analysis of observational studies | 2014 | PLoS ONE | 9 | 6 | 10.1371/journal.pone.0099050 | |  |
| Fernando M.E.; Seneviratne R.M.; Tan Y.M.; Lazzarini P.A.; Sangla K.S.; Cunningham M.; Buttner P.G.; Golledge J. | Intensive versus conventional glycaemic control for treating diabetic foot ulcers | 2016 | Cochrane Database of Systematic Reviews | 2016 | 1 | 10.1002/14651858.CD010764.pub2 | |  |
| Frescos N.; Copnell B. | Podiatrists' views of assessment and management of pain in diabetes-related foot ulcers: A focus group study | 2020 | Journal of Foot and Ankle Research | 13 | 1 | 10.1186/s13047-020-00399-8 | |  |
| Frescos N.; Stopher L.; Jansen S.; Kaminski M.R. | The financial burden of diabetes-related foot disease in Australia: a systematic review | 2023 | Journal of Foot and Ankle Research | 16 | 1 | 10.1186/s13047-023-00688-y | |  |
| Gardiner M.; Vicaretti M.; Sparks J.; Bansal S.; Bush S.; Liu M.; Darling A.; Harry E.; Burke C.M. | A longitudinal study of the diabetic skin and wound microbiome | 2017 | PeerJ | 2017 | 7 | 10.7717/peerj.3543 | |  |
| Golledge J.; Fernando M.E.; Alahakoon C.; Lazzarini P.A.; aan de Stegge W.B.; van Netten J.J.; Bus S.A. | Efficacy of at home monitoring of foot temperature for risk reduction of diabetes-related foot ulcer: A meta-analysis | 2022 | Diabetes/Metabolism Research and Reviews | 38 | 6 | 10.1002/dmrr.3549 | |  |
| Golledge J.; Singh T.P. | Systematic review and meta-analysis of clinical trials examining the effect of hyperbaric oxygen therapy in people with diabetes-related lower limb ulcers | 2019 | Diabetic Medicine | 36 | 7 | 10.1111/dme.13975 | |  |
| Graham K.; Siatis C.M.; Gunn K.M.; Ong E.; Loughry C.; McMillan N.; Fitridge R. | The experiences of health workers using telehealth services for diabetes-related foot complications: a qualitative exploration | 2023 | Journal of Foot and Ankle Research | 16 | 1 | 10.1186/s13047-023-00645-9 | |  |
| Griffiths D.A.; Kaminski M.R. | Duration of total contact casting for resolution of acute Charcot foot: a retrospective cohort study | 2021 | Journal of Foot and Ankle Research | 14 | 1 | 10.1186/s13047-021-00477-5 | |  |
| Gunton J.E.; Girgis C.M.; Lau T.; Vicaretti M.; Begg L.; Flood V. | Vitamin C improves healing of foot ulcers: A randomised, double-blind, placebo-controlled trial | 2021 | British Journal of Nutrition | 126 | 10 | 10.1017/S0007114520003815 | |  |
| Haji Zaine N.; Hitos K.; Vicaretti M.; Fletcher J.P.; Begg L.; Burns J. | Characteristics of non-diabetic foot ulcers in Western Sydney, Australia | 2016 | Journal of Foot and Ankle Research | 9 | 1 | 10.1186/s13047-016-0137-6 | |  |
| Hamilton E.J.; Davis W.A.; Baba M.; Davis T.M.E. | Temporal trends in minor and major lower extremity amputation in people with type 2 diabetes: The Fremantle Diabetes Study | 2023 | Diabetes and Vascular Disease Research | 20 | 1 | 10.1177/14791641231154162 | |  |
| Hamilton E.J.; Davis W.A.; Siru R.; Baba M.; Norman P.E.; Davis T.M.E. | Temporal trends in incident hospitalization for diabetes-related foot ulcer in type 2 diabetes: The fremantle diabetes study | 2021 | Diabetes Care | 44 | 3 | 10.2337/dc20-1743 | |  |
| Hand R.; Manning L.; Ritter J.C.; Norman P.; Lamb L.; Makepeace A.; Sankhesara D.; Hamilton E.; Ingram P. | Antimicrobial stewardship opportunities among inpatients with diabetic foot infections: microbiology results from a tertiary hospital multidisciplinary unit | 2019 | Internal Medicine Journal | 49 | 4 | 10.1111/imj.14251 | |  |
| Hayes O.G.; Vangaveti V.N.; Malabu U.H. | Serum procollagen type 1 N propeptide: A novel diagnostic test for diabetic foot osteomyelitis – A case–control study | 2018 | Journal of Research in Medical Sciences | 23 | 5 | 10.4103/jrms.JRMS_810_16 | |  |
| Henshaw F.R.; Bolton T.; Nube V.; Hood A.; Veldhoen D.; Pfrunder L.; McKew G.L.; MacLeod C.; McLennan S.V.; Twigg S.M. | Topical application of the bee hive protectant propolis is well tolerated and improves human diabetic foot ulcer healing in a prospective feasibility study | 2014 | Journal of Diabetes and its Complications | 28 | 6 | 10.1016/j.jdiacomp.2014.07.012 | |  |
| Henshaw F.R.; Bostan L.E.; Worsley P.R.; Bader D.L. | Evaluating the effects of sedentary behaviour on plantar skin health in people with diabetes | 2020 | Journal of Tissue Viability | 29 | 4 | 10.1016/j.jtv.2020.09.001 | |  |
| Henshaw F.R.; Brennan L.; MacMillan F. | Perceptions of hyperbaric oxygen therapy among podiatrists practicing in high-risk foot clinics | 2018 | International Wound Journal | 15 | 3 | 10.1111/iwj.12874 | |  |
| Heravi F.S.; Zakrzewski M.; Aboulkheyr Estarabadi H.; Vickery K.; Hu H. | Evaluation of Host Immune Response in Diabetic Foot Infection Tissues Using an RNA Sequencing-Based Approach | 2021 | Frontiers in Microbiology | 12 |  | 10.3389/fmicb.2021.613697 | |  |
| Heravi F.S.; Zakrzewski M.; Vickery K.; Malone M.; Hu H. | Metatranscriptomic Analysis Reveals Active Bacterial Communities in Diabetic Foot Infections | 2020 | Frontiers in Microbiology | 11 |  | 10.3389/fmicb.2020.01688 | |  |
| Heuch L.; Streak Gomersall J. | Effectiveness of offloading methods in preventing primary diabetic foot ulcers in adults with diabetes: a systematic review | 2016 | JBI database of systematic reviews and implementation reports | 14 | 7 | 10.11124/JBISRIR-2016-003013 | |  |
| Houghton V.J.; Bower V.M.; Chant D.C. | Is an increase in skin temperature predictive of neuropathic foot ulceration in people with diabetes? A systematic review and meta-analysis | 2013 | Journal of Foot and Ankle Research | 6 | 1 | 10.1186/1757-1146-6-31 | |  |
| Iseli R.K.; Lee E.K.; Lewis E.; Duncan G.; Maier A.B. | Foot disease and physical function in older adults: A systematic review and meta-analysis | 2021 | Australasian Journal on Ageing | 40 | 1 | 10.1111/ajag.12892 | |  |
| Ismail I.; Dhanapathy A.; Gandhi A.; Kannan S. | Diabetic foot complications in a secondary foot hospital: A clinical audit | 2015 | Australasian Medical Journal | 8 | 4 | 10.4066/AMJ.2015.2274 | |  |
| Jelinek H.F.; Prinz M.; Wild T. | A digital assessment and documentation tool evaluated for daily podiatric wound practice | 2013 | Wounds | 25 | 1 |  |  | |
| Jelinek H.F.; Thompson L.; Tinley P. | Diagnosis of peripheral vascular disease for diabetic foot risk assessment | 2014 | Wound Medicine | 4 |  | 10.1016/j.wndm.2014.02.002 | |  |
| Jelinek H.F.; Wilding C.; Tinley P. | An innovative multi-disciplinary diabetes complications screening program in a rural community: A description and preliminary results of the screening | 2006 | Australian Journal of Primary Health | 12 | 1 | 10.1071/py06003 | |  |
| Jessup R.L.; Spring A.A.; Grollo A. | Current practice in the assessment and management of acute diabetes-related foot complications. | 2007 | Australian health review : a publication of the Australian Hospital Association | 31 | 2 | 10.1071/AH070217 | |  |
| Jeyaraman K.; Berhane T.; Hamilton M.; Chandra A.P.; Falhammar H. | Mortality in patients with diabetic foot ulcer: A retrospective study of 513 cases from a single Centre in the Northern Territory of Australia | 2019 | BMC Endocrine Disorders | 19 | 1 | 10.1186/s12902-018-0327-2 | |  |
| Jeyaraman K.; Berhane T.; Hamilton M.; Chandra A.P.; Falhammar H. | Amputations in patients with diabetic foot ulcer: a retrospective study from a single centre in the Northern Territory of Australia | 2019 | ANZ Journal of Surgery | 89 | 7 | 10.1111/ans.15351 | |  |
| Jia L.; Parker C.N.; Parker T.J.; Kinnear E.M.; Derhy P.H.; Alvarado A.M.; Huygens F.; Lazzarini P.A. | Incidence and risk factors for developing infection in patients presenting with uninfected diabetic foot ulcers | 2017 | PLoS ONE | 12 | 5 | 10.1371/journal.pone.0177916 | |  |
| Johani K.; Fritz B.G.; Bjarnsholt T.; Lipsky B.A.; Jensen S.O.; Yang M.; Dean A.; Hu H.; Vickery K.; Malone M. | Understanding the microbiome of diabetic foot osteomyelitis: insights from molecular and microscopic approaches | 2019 | Clinical Microbiology and Infection | 25 | 3 | 10.1016/j.cmi.2018.04.036 | |  |
| Johani K.; Malone M.; Jensen S.; Gosbell I.; Dickson H.; Hu H.; Vickery K. | Microscopy visualisation confirms multi-species biofilms are ubiquitous in diabetic foot ulcers | 2017 | International Wound Journal | 14 | 6 | 10.1111/iwj.12777 | |  |
| Johnson N.A.; Barwick A.L.; Searle A.; Spink M.J.; Twigg S.M.; Chuter V.H. | Self-reported physical activity in community-dwelling adults with diabetes and its association with diabetes complications | 2019 | Journal of Diabetes and its Complications | 33 | 1 | 10.1016/j.jdiacomp.2018.10.017 | |  |
| Joseph S.; Munshi B.; Agarini R.; Kwok R.C.H.; Green D.J.; Jansen S. | Near infrared spectroscopy in peripheral artery disease and the diabetic foot: A systematic review | 2022 | Diabetes/Metabolism Research and Reviews | 38 | 7 | 10.1002/dmrr.3571 | |  |
| Kaczmarek T.; Van Netten J.J.; Lazzarini P.A.; Kavanagh D. | Effects of training podiatrists to use imagery-based motivational interviewing when treating people with diabetes-related foot disease: a mixed-methods pilot study | 2021 | Journal of Foot and Ankle Research | 14 | 1 | 10.1186/s13047-021-00451-1 | |  |
| Khunkaew S.; Fernandez R.; Sim J. | Health-related quality of life among adults living with diabetic foot ulcers: a meta-analysis | 2019 | Quality of Life Research | 28 | 6 | 10.1007/s11136-018-2082-2 | |  |
| Kiburg K.V.; Galligan A.; Sundararajan V.; MacIsaac R.J. | Temporal trends in non-traumatic lower extremity amputations (LEAs) and their association with 12-month mortality in people with diabetes, 2004–2016 | 2022 | Journal of Diabetes and its Complications | 36 | 7 | 10.1016/j.jdiacomp.2022.108221 | |  |
| Kuang B.; Pena G.; Szpak Z.; Edwards S.; Battersby R.; Cowled P.; Dawson J.; Fitridge R. | Assessment of a smartphone-based application for diabetic foot ulcer measurement | 2021 | Wound Repair and Regeneration | 29 | 3 | 10.1111/wrr.12905 | |  |
| Kueh Y.C.; Morris T.; Borkoles E.; Shee H. | Modelling of diabetes knowledge, attitudes, self-management, and quality of life: A cross-sectional study with an Australian sample | 2015 | Health and Quality of Life Outcomes | 13 | 1 | 10.1186/s12955-015-0303-8 | |  |
| Kurowski J.R.; Nedkoff L.; Schoen D.E.; Knuiman M.; Norman P.E.; Briffa T.G. | Temporal trends in initial and recurrent lower extremity amputations in people with and without diabetes in Western Australia from 2000 to 2010 | 2015 | Diabetes Research and Clinical Practice | 108 | 2 | 10.1016/j.diabres.2015.02.008 | |  |
| Lafontaine N.; Jolley J.; Kyi M.; King S.; Iacobaccio L.; Staunton E.; Wilson B.; Seymour C.; Rogasch S.; Wraight P. | Prospective randomised placebo-controlled trial assessing the efficacy of silver dressings to enhance healing of acute diabetes-related foot ulcers | 2023 | Diabetologia | 66 | 4 | 10.1007/s00125-022-05855-7 | |  |
| Lanting S.; Way K.; Sabag A.; Sultana R.; Gerofi J.; Johnson N.; Baker M.; Keating S.; Caterson I.; Twigg S.; Chuter V. | The Efficacy of Exercise Training for Cutaneous Microvascular Reactivity in the Foot in People with Diabetes and Obesity: Secondary Analyses from a Randomized Controlled Trial | 2022 | Journal of Clinical Medicine | 11 | 17 | 10.3390/jcm11175018 | |  |
| Lanting S.M.; Barwick A.L.; Twigg S.M.; Johnson N.A.; Baker M.K.; Chiu S.K.; Caterson I.D.; Chuter V.H. | Post-occlusive reactive hyperaemia of skin microvasculature and foot complications in type 2 diabetes | 2017 | Journal of Diabetes and its Complications | 31 | 8 | 10.1016/j.jdiacomp.2017.05.005 | |  |
| Lanting S.M.; Chan T.L.; Casey S.L.; Peterson B.J.; Chuter V.H. | Cutaneous microvascular reactivity in Charcot neuroarthropathy: a systematic review and meta-analysis | 2022 | Journal of Foot and Ankle Research | 15 | 1 | 10.1186/s13047-022-00522-x | |  |
| Lanting S.M.; Twigg S.M.; Johnson N.A.; Baker M.K.; Caterson I.D.; Chuter V.H. | Non-invasive lower limb small arterial measures co-segregate strongly with foot complications in people with diabetes | 2017 | Journal of Diabetes and its Complications | 31 | 3 | 10.1016/j.jdiacomp.2016.11.010 | |  |
| Lasschuit J.W.J.; Featherston J.; Tonks K.T.T. | Reliability of a Three-Dimensional Wound Camera and Correlation With Routine Ruler Measurement in Diabetes-Related Foot Ulceration | 2020 | Journal of Diabetes Science and Technology | 15 | 6 | 10.1177/1932296820974654 | |  |
| Lasschuit J.W.J.; Greenfield J.R.; Tonks K.T.T. | Contribution of peripheral neuropathy to poor bone health in the feet of people with type 2 diabetes mellitus | 2022 | Acta Diabetologica | 59 | 2 | 10.1007/s00592-021-01803-w | |  |
| Lawrence E.; Li F. | Foot burns and diabetes: A retrospective study | 2015 | Burns and Trauma | 3 | 1 | 10.1186/s41038-015-0024-6 | |  |
| Lawrence S.M.; Wraight P.R.; Campbell D.A.; Colman P.G. | Assessment and management of inpatients with acute diabetes-related foot complications: Room for improvement | 2004 | Internal Medicine Journal | 34 | 5 | 10.1111/j.1444-0903.2004.00590.x | |  |
| Lazzarini P.A.; Cramb S.M.; Golledge J.; Morton J.I.; Magliano D.J.; Van Netten J.J. | Global trends in the incidence of hospital admissions for diabetes-related foot disease and amputations: a review of national rates in the 21st century | 2023 | Diabetologia | 66 | 2 | 10.1007/s00125-022-05845-9 | |  |
| Lazzarini P.A.; Hurn S.E.; Fernando M.E.; Jen S.D.; Kuys S.S.; Kamp M.C.; Reed L.F. | Prevalence of foot disease and risk factors in general inpatient populations: A systematic review and meta-analysis | 2015 | BMJ Open | 5 | 11 | 10.1136/bmjopen-2015-008544 | |  |
| Lazzarini P.A.; Hurn S.E.; Kuys S.S.; Kamp M.C.; Ng V.; Thomas C.; Jen S.; Kinnear E.M.; D'Emden M.C.; Reed L. | Direct inpatient burden caused by foot-related conditions: A multisite point-prevalence study | 2016 | BMJ Open | 6 | 6 | 10.1136/bmjopen-2015-010811 | |  |
| Lazzarini P.A.; Hurn S.E.; Kuys S.S.; Kamp M.C.; Ng V.; Thomas C.; Jen S.; Wills J.; Kinnear E.M.; d'Emden M.C.; Reed L.F. | The silent overall burden of foot disease in a representative hospitalised population | 2017 | International Wound Journal | 14 | 4 | 10.1111/iwj.12683 | |  |
| Lazzarini P.A.; Hurn S.E.; Kuys S.S.; Kamp M.C.; Ng V.; Thomas C.; Jen S.; Wills J.; Kinnear E.M.; D'Emden M.C.; Reed L.F. | Foot Complications in a Representative Australian Inpatient Population | 2017 | Journal of Diabetes Research | 2017 |  | 10.1155/2017/4138095 | |  |
| Lazzarini P.A.; Jarl G.; Gooday C.; Viswanathan V.; Caravaggi C.F.; Armstrong D.G.; Bus S.A. | Effectiveness of offloading interventions to heal foot ulcers in persons with diabetes: a systematic review | 2020 | Diabetes/Metabolism Research and Reviews | 36 | S1 | 10.1002/dmrr.3275 | |  |
| Lazzarini P.A.; Mackenroth E.L.; Régo P.M.; Boyle F.M.; Jen S.; Kinnear E.M.; PerryHaines G.M.; Kamp M. | Is simulation training effective in increasing podiatrists' confidence in foot ulcer management? | 2011 | Journal of Foot and Ankle Research | 4 | 1 | 10.1186/1757-1146-4-16 | |  |
| Lazzarini P.A.; Ng V.; Kinnear E.M.; Kamp M.C.; Kuys S.S.; Hurst C.; Reed L.F. | The Queensland high risk foot form (QHRFF) - is it a reliable and valid clinical research tool for foot disease? | 2014 | Journal of Foot and Ankle Research | 7 | 1 | 10.1186/1757-1146-7-7 | |  |
| Lazzarini P.A.; O'Rourke S.R.; Russell A.W.; Clark D.; Kuys S.S. | What are the key conditions associated with lower limb amputations in a major Australian teaching hospital? | 2012 | Journal of Foot and Ankle Research | 5 | 1 | 10.1186/1757-1146-5-12 | |  |
| Lazzarini P.A.; O'Rourke S.R.; Russell A.W.; Derhy P.H.; Kamp M.C. | Reduced incidence of foot-related hospitalisation and amputation amongst persons with diabetes in Queensland, Australia | 2015 | PLoS ONE | 10 | 6 | 10.1371/journal.pone.0130609 | |  |
| Lee A.S.; Twigg S.M.; Flack J.R. | Metabolic syndrome in type 1 diabetes and its association with diabetes complications | 2021 | Diabetic Medicine | 38 | 2 | 10.1111/dme.14376 | |  |
| Lee J.; Mashayamombe M.; Walsh T.P.; Kuang B.K.P.; Pena G.N.; Vreugde S.; Cooksley C.; Carda-Diéguez M.; Mira A.; Jesudason D.; Fitridge R.; Zilm P.S.; Dawson J.; Kidd S.P. | The bacteriology of diabetic foot ulcers and infections and incidence of Staphylococcus aureus Small Colony Variants | 2023 | Journal of Medical Microbiology | 72 | 6 | 10.1099/jmm.0.001716 | |  |
| Lee M.; van Netten J.J.; Sheahan H.; Lazzarini P.A. | Moderate-to-Vigorous-Intensity Physical Activity Observed in People With Diabetes-Related Foot Ulcers Over a One-Week Period | 2019 | Journal of Diabetes Science and Technology | 13 | 5 | 10.1177/1932296819848735 | |  |
| Leo J.; Nicholls K. | Foot screening in a dialysis unit - A pilot educational project | 2015 | Renal Society of Australasia Journal | 11 | 3 |  |  | |
| Linton C.; Searle A.; Hawke F.; Tehan P.E.; Chuter V. | Nature and extent of outpatient podiatry service utilisation in people with diabetes undergoing minor foot amputations: a retrospective clinical audit | 2021 | Journal of Foot and Ankle Research | 14 | 1 | 10.1186/s13047-020-00445-5 | |  |
| Linton C.; Searle A.; Hawke F.; Tehan P.E.; Sebastian M.; Chuter V. | Do toe blood pressures predict healing after minor lower limb amputation in people with diabetes? A systematic review and meta-analysis | 2020 | Diabetes and Vascular Disease Research | 17 | 3 | 10.1177/1479164120928868 | |  |
| Liu Y.; Brooks B.; McCosker S.; Molyneaux L.; Yue D.K. | How do we distinguish loss of vibration sensation due to neuropathy from that due to ageing? | 2013 | Practical Diabetes | 30 | 5 | 10.1002/pdi.1773 | |  |
| Longfield M.S.G.; Lourdesamy J.; Min D.; Twigg S.M. | Do diabetes-related foot ulcer wound fluid measures have clinical utility as biomarkers for healing? A systematic review | 2023 | Journal of Wound Care | 32 |  | 10.12968/jowc.2023.32.Sup4a.xlvii | |  |
| Lynar S.A.; Robinson C.H.; Boutlis C.S.; Commons R.J. | Risk factors for mortality in patients with diabetic foot infections: a prospective cohort study | 2019 | Internal Medicine Journal | 49 | 7 | 10.1111/imj.14184 | |  |
| Macdonald E.M.; Perrin B.M.; Cleeland L.; Kingsley M.I.C. | Podiatrist-delivered health coaching to facilitate the use of a smart insole to support foot health monitoring in people with diabetes-related peripheral neuropathy | 2021 | Sensors | 21 | 12 | 10.3390/s21123984 | |  |
| Macdonald E.M.; Perrin B.M.; Hyett N.; Kingsley M.I.C. | Factors influencing behavioural intention to use a smart shoe insole in regionally based adults with diabetes: A mixed methods study | 2019 | Journal of Foot and Ankle Research | 12 | 1 | 10.1186/s13047-019-0340-3 | |  |
| Malone M.; Bowling F.L.; Gannass A.; Jude E.B.; Boulton A.J.M. | Deep wound cultures correlate well with bone biopsy culture in diabetic foot osteomyelitis | 2013 | Diabetes/Metabolism Research and Reviews | 29 | 7 | 10.1002/dmrr.2425 | |  |
| Malone M.; Erasmus A.; Schwarzer S.; Lau N.S.; Ahmad M.; Dickson H.G. | Utilisation of the 2019 IWGDF diabetic foot infection guidelines to benchmark practice and improve the delivery of care in persons with diabetic foot infections | 2021 | Journal of Foot and Ankle Research | 14 | 1 | 10.1186/s13047-021-00448-w | |  |
| Malone M.; Fritz B.G.; Vickery K.; Schwarzer S.; Sharma V.; Biggs N.; Radzieta M.; Jeffries T.T.; Dickson H.G.; Jensen S.O.; Bjarnsholt T. | Analysis of proximal bone margins in diabetic foot osteomyelitis by conventional culture, DNA sequencing and microscopy | 2019 | APMIS | 127 | 10 | 10.1111/apm.12986 | |  |
| Malone M.; Johani K.; Jensen S.O.; Gosbell I.B.; Dickson H.G.; Hu H.; Vickery K. | Next Generation DNA Sequencing of Tissues from Infected Diabetic Foot Ulcers | 2017 | EBioMedicine | 21 |  | 10.1016/j.ebiom.2017.06.026 | |  |
| Malone M.; Johani K.; Jensen S.O.; Gosbell I.B.; Dickson H.G.; McLennan S.; Hu H.; Vickery K. | Effect of cadexomer iodine on the microbial load and diversity of chronic non-healing diabetic foot ulcers complicated by biofilm in vivo | 2017 | Journal of Antimicrobial Chemotherapy | 72 | 7 | 10.1093/jac/dkx099 | |  |
| Malone M.; Radzieta M.; Peters T.J.; Dickson H.G.; Schwarzer S.; Jensen S.O.; Lavery L.A. | Host-microbe metatranscriptome reveals differences between acute and chronic infections in diabetes-related foot ulcers | 2022 | APMIS | 130 | 12 | 10.1111/apm.13200 | |  |
| Malone M.; Radzieta M.; Schwarzer S.; Jensen S.O.; Lavery L.A. | Efficacy of a topical concentrated surfactant gel on microbial communities in non-healing diabetic foot ulcers with chronic biofilm infections: A proof-of-concept study | 2021 | International Wound Journal | 18 | 4 | 10.1111/iwj.13546 | |  |
| Malone M.; Radzieta M.; Schwarzer S.; Walker A.; Bradley J.; Jensen S.O. | In vivo observations of biofilm adhering to a dialkylcarbamoyl chloride-coated mesh dressing when applied to diabetes-related foot ulcers: A proof of concept study | 2023 | International Wound Journal | 20 | 6 | 10.1111/iwj.14054 | |  |
| Malone M.; Schwarzer S.; Radzieta M.; Jeffries T.; Walsh A.; Dickson H.G.; Micali G.; Jensen S.O. | Effect on total microbial load and community composition with two vs six-week topical Cadexomer Iodine for treating chronic biofilm infections in diabetic foot ulcers | 2019 | International Wound Journal | 16 | 6 | 10.1111/iwj.13219 | |  |
| Malone M.; Schwarzer S.; Walsh A.; Xuan W.; Al Gannass A.; Dickson H.G.; Bowling F.L. | Monitoring wound progression to healing in diabetic foot ulcers using three-dimensional wound imaging | 2020 | Journal of Diabetes and its Complications | 34 | 2 | 10.1016/j.jdiacomp.2019.107471 | |  |
| Malone M.; West D.; Xuan W.; Lau N.S.; Maley M.; Dickson H.G. | Outcomes and cost minimisation associated with outpatient parenteral antimicrobial therapy (OPAT) for foot infections in people with diabetes | 2015 | Diabetes/Metabolism Research and Reviews | 31 | 6 | 10.1002/dmrr.2651 | |  |
| Manewell S.M.; Aitken S.J.; Nube V.L.; Crawford A.M.; Constantino M.I.; Twigg S.M.; Menz H.B.; Sherrington C.; Paul S.S. | Timing of minor and major amputation in patients with diabetes-related foot ulceration admitted to two public tertiary referral hospitals in Australia | 2023 | ANZ Journal of Surgery | 93 | 6 | 10.1111/ans.18224 | |  |
| Manning L.; Ferreira I.B.; Gittings P.; Hiew J.; Ryan E.; Baba M.; Raby E.; Carville K.; Norman P.E.; Davis W.A.; Wood F.; Hamilton E.J.; Ritter J.C. | Wound healing with “spray-on” autologous skin grafting (ReCell) compared with standard care in patients with large diabetes-related foot wounds: an open-label randomised controlled trial | 2022 | International Wound Journal | 19 | 3 | 10.1111/iwj.13646 | |  |
| Manning L.; Hamilton E.J.; Raby E.; Norman P.E.; Davis W.; Wood F.; Carville K.; Baba M.; Hiew J.; Ryan E.; Ferreira I.; Gittings P.; Ritter J.C. | Spray on skin for diabetic foot ulcers: An open label randomised controlled trial | 2019 | Journal of Foot and Ankle Research | 12 | 1 | 10.1186/s13047-019-0362-x | |  |
| Maple-Brown L.J.; Cunningham J.; Zinman B.; Mamakeesick M.; Harris S.B.; Connelly P.W.; Shaw J.; O'Dea K.; Hanley A.J. | Cardiovascular disease risk profile and microvascular complications of diabetes: Comparison of Indigenous cohorts with diabetes in Australia and Canada | 2012 | Cardiovascular Diabetology | 11 |  | 10.1186/1475-2840-11-30 | |  |
| Martin J.D.; Delbridge L.; Reeve T.S.; Clagett G.P. | Radical treatment of mal perforans in diabetic patients with arterial insufficiency | 1990 | Journal of Vascular Surgery | 12 | 3 | 10.1016/0741-5214(90)90146-2 | |  |
| Matricciani L.; Talbot K.; Jones S. | Safety and efficacy of tinea pedis and onychomycosis treatment in people with diabetes: A systematic review | 2011 | Journal of Foot and Ankle Research | 4 | 1 | 10.1186/1757-1146-4-26 | |  |
| Matthews D.R.; Li Q.; Perkovic V.; Mahaffey K.W.; de Zeeuw D.; Fulcher G.; Desai M.; Hiatt W.R.; Nehler M.; Fabbrini E.; Kavalam M.; Lee M.; Neal B. | Effects of canagliflozin on amputation risk in type 2 diabetes: the CANVAS Program | 2019 | Diabetologia | |  | 10.1007/s00125-019-4839-8 | |  |
| McCosker L.; Tulleners R.; Cheng Q.; Rohmer S.; Pacella T.; Graves N.; Pacella R. | Chronic wounds in Australia: A systematic review of key epidemiological and clinical parameters | 2019 | International Wound Journal | 16 | 1 | 10.1111/iwj.12996 | |  |
| McDermott R.A.; Tulip F.; Schmidt B. | Diabetes care in remote northern Australian indigenous communities | 2004 | Medical Journal of Australia | 180 | 10 | 10.5694/j.1326-5377.2004.tb06055.x | |  |
| Mcdonald S.; Sharpe L.; Blaszczynski A. | The psychosocial impact associated with diabetes-related amputation | 2014 | Diabetic Medicine | 31 | 11 | 10.1111/dme.12474 | |  |
| McDonogh C.; Nube V.L.; Frank G.; Twigg S.M.; Penkala S.; Holloway S.; Snyder R. | Does in-shoe pressure analysis to assess and modify medical grade footwear improve patient adherence and understanding? A mixed methods study | 2022 | Journal of Foot and Ankle Research | 15 | 1 | 10.1186/s13047-022-00600-0 | |  |
| McGill M.; Constantino M.; Yue D.K. | Integrating telemedicine into a National Diabetes Footcare Network | 2000 | Practical Diabetes International | 17 | 7 | 10.1002/1528-252X(200010)17:7<235::AID-PDI101>3.0.CO;2-H | |  |
| McGill M.; Molyneaux L.; Yue D.K. | Use of the Semmes-Weinstein 5.07/10 gram monofilament: The long and the short of it | 1998 | Diabetic Medicine | 15 | 7 | 10.1002/(SICI)1096-9136(199807)15:7<615::AID-DIA641>3.0.CO;2-P | |  |
| McGill M.; Molyneaux L.; Yue D.K. | Which diabetic patients should receive podiatry care? An objective analysis | 2005 | Internal Medicine Journal | 35 | 8 | 10.1111/j.1445-5994.2005.00880.x | |  |
| McGill M.; Molyneaux L.M.; Yue D.K.; Turtle J.R. | A Single Visit Diabetes Complication Assessment Service: a Complement to Diabetes Management at the Primary Care Level | 1993 | Diabetic Medicine | 10 | 4 | 10.1111/j.1464-5491.1993.tb00082.x | |  |
| McIllhatton A.; Lanting S.; Lambkin D.; Leigh L.; Casey S.; Chuter V. | Reliability of recommended non-invasive chairside screening tests for diabetes-related peripheral neuropathy: A systematic review with meta-analyses | 2021 | BMJ Open Diabetes Research and Care | 9 | 2 | 10.1136/bmjdrc-2021-002528 | |  |
| Michailidis L.; Bergin S.M.; Haines T.P.; Williams C.M. | Healing rates in diabetes-related foot ulcers using low frequency ultrasonic debridement versus non-surgical sharps debridement: A randomised controlled trial ACTRN12612000490875 ACTRN 11 Medical and Health Sciences 1103 Clinical Sciences | 2018 | BMC Research Notes | 11 | 1 | 10.1186/s13104-018-3841-4 | |  |
| Michailidis L.; Bergin S.M.; Haines T.P.; Williams C.M. | A systematic review to compare the effect of low-frequency ultrasonic versus nonsurgical sharp debridement on the healing rate of chronic diabetes-related foot ulcers | 2018 | Ostomy Wound Management | 64 | 9 | 10.25270/owm.2018.9.3946 | |  |
| Min D.; Nube V.; Tao A.; Yuan X.; Williams P.F.; Brooks B.A.; Wong J.; Twigg S.M.; McLennan S.V. | Monocyte phenotype as a predictive marker for wound healing in diabetes-related foot ulcers | 2021 | Journal of Diabetes and its Complications | 35 | 5 | 10.1016/j.jdiacomp.2021.107889 | |  |
| Morona J.K.; Buckley E.S.; Jones S.; Reddin E.A.; Merlin T.L. | Comparison of the clinical effectiveness of different off-loading devices for the treatment of neuropathic foot ulcers in patients with diabetes: A systematic review and meta-analysis | 2013 | Diabetes/Metabolism Research and Reviews | 29 | 3 | 10.1002/dmrr.2386 | |  |
| Morrison T.; Jones S.; Causby R.S.; Thoirs K. | Can ultrasound measures of intrinsic foot muscles and plantar soft tissues predict future diabetes-related foot disease? A systematic review | 2018 | PLoS ONE | 13 | 6 | 10.1371/journal.pone.0199055 | |  |
| Morrison T.; Jones S.; Causby R.S.; Thoirs K. | Reliability of ultrasound in evaluating the plantar skin and fat pad of the foot in the setting of diabetes | 2021 | PLoS ONE | 16 | 9-Sep | 10.1371/journal.pone.0257790 | |  |
| Motaganahalli S.; Batrouney A.; Perera D.; Vogrin S.; Trubiano J.A. | Retrospective study of outcomes of short versus long duration of antibiotic therapy for residual osteomyelitis in surgically resected diabetic foot infection | 2023 | Journal of Antimicrobial Chemotherapy | 78 | 1 | 10.1093/jac/dkac390 | |  |
| Mullan L.; Wynter K.; Driscoll A.; Rasmussen B. | Preventative and early intervention diabetes-related foot care practices in primary care | 2020 | Australian Journal of Primary Health | 26 | 2 | 10.1071/PY19183 | |  |
| Mullan L.; Wynter K.; Driscoll A.; Rasmussen B. | Prioritisation of diabetes-related footcare amongst primary care healthcare professionals | 2020 | Journal of Clinical Nursing | 29 | 23-24 | 10.1111/jocn.15506 | |  |
| Mullan L.; Wynter K.; Driscoll A.; Rasmussen B. | Implementation strategies to overcome barriers to diabetes-related footcare delivery in primary care: A qualitative study | 2021 | Australian Journal of Primary Health | 27 | 4 | 10.1071/PY20241 | |  |
| Mullan L.; Wynter K.; Driscoll A.; Rasmussen B. | Barriers and enablers to providing preventative and early intervention diabetes-related foot care: A qualitative study of primary care healthcare professionals' perceptions | 2021 | Australian Journal of Primary Health | 27 | 4 | 10.1071/PY20235 | |  |
| Nejatian M.M.; Sobhi S.; Sanchez B.N.; Linn K.; Manning L.; Soh S.-C.; Hiew J.; Ritter J.C.; Yeap B.B.; Hamilton E.J. | Reduction in femoral neck and total hip bone mineral density following hospitalisation for diabetes-related foot ulceration | 2021 | Scientific Reports | 11 | 1 | 10.1038/s41598-021-02233-y | |  |
| Netten J.J.V.; Clark D.; Lazzarini P.A.; Janda M.; Reed L.F. | The validity and reliability of remote diabetic foot ulcer assessment using mobile phone images | 2017 | Scientific Reports | 7 | 1 | 10.1038/s41598-017-09828-4 | |  |
| Nguyen M.; Wong D.; Barson E.; Staunton E.T.; Fisher C.A. | Psychological and Cognitive Barriers to Diabetes-Related Foot Complication Treatment: Clinicians’ Perspectives | 2022 | International Journal of Lower Extremity Wounds | 21 | 4 | 10.1177/1534734620983181 | |  |
| Nguyen T.P.L.; Edwards H.; Do T.N.D.; Finlayson K. | Effectiveness of a theory-based foot care education program (3STEPFUN) in improving foot self-care behaviours and foot risk factors for ulceration in people with type 2 diabetes | 2019 | Diabetes Research and Clinical Practice | 152 |  | 10.1016/j.diabres.2019.05.003 | |  |
| Nube V.L.; Alison J.A.; Twigg S.M. | Frequency of sharp wound debridement in the management of diabetes-related foot ulcers: exploring current practice | 2021 | Journal of Foot and Ankle Research | 14 | 1 | 10.1186/s13047-021-00489-1 | |  |
| Nubé V.L.; Molyneaux L.; Bolton T.; Clingan T.; Palmer E.; Yue D.K. | The use of felt deflective padding in the management of plantar hallux and forefoot ulcers in patients with diabetes | 2006 | Foot | 16 | 1 | 10.1016/j.foot.2005.11.005 | |  |
| Nubé V.L.; Molyneaux L.; Yue D.K. | Biomechanical risk factors associated with neuropathic ulceration of the hallux in people with diabetes mellitus | 2006 | Journal of the American Podiatric Medical Association | 96 | 3 | 10.7547/0960189 | |  |
| Nube V.L.; White J.M.; Brewer K.; Veldhoen D.; Meler C.; Frank G.; Carroll K.; Featherston J.; Batchelor J.; Gebski V.; Alison J.A.; Twigg S.M. | A randomized trial comparing weekly with every second week sharp debridement in people with diabetes-related foot ulcers shows similar healing outcomes: Potential benefit to resource utilization | 2021 | Diabetes Care | 44 | 12 | 10.2337/dc21-1454 | |  |
| Ogrin R.; Viswanathan R.; Aylen T.; Wallace F.; Scott J.; Kumar D. | Co-design of an evidence-based health education diabetes foot app to prevent serious foot complications: a feasibility study | 2018 | Practical Diabetes | 35 | 6 | 10.1002/pdi.2197 | |  |
| Ong E.K.M.; Fryer C.; Graham K.; Causby R.S. | Investigating the experience of receiving podiatry care in a tertiary care hospital clinic for people with diabetes related foot ulcers | 2022 | Journal of Foot and Ankle Research | 15 | 1 | 10.1186/s13047-022-00556-1 | |  |
| O'Rourke I.; Heard S.; Treacy J.; Gruen R.; Whitbread C. | Risks to feet in the top end: Outcomes of diabetic foot complications | 2002 | ANZ Journal of Surgery | 72 | 4 | 10.1046/j.1445-2197.2002.02367.x | |  |
| O'Rourke S.; Steffen C.; Raulli A.; Tulip F. | Diabetic major amputation in far north Queensland 1998-2008: What is the Gap for Indigenous patients? | 2013 | Australian Journal of Rural Health | 21 | 5 | 10.1111/ajr.12044 | |  |
| Palaya J.; Pearson S.; Nash T. | Perception of social support in individuals living with a diabetic foot: A qualitative study | 2018 | Diabetes Research and Clinical Practice | 146 |  | 10.1016/j.diabres.2018.10.016 | |  |
| Pang B.; Shah P.M.; Manning L.; Ritter J.C.; Hiew J.; Hamilton E.J. | Management of diabetes-related foot disease in the outpatient setting during the COVID-19 pandemic | 2021 | Internal Medicine Journal | 51 | 7 | 10.1111/imj.15392 | |  |
| Parker C.N.; Shuter P.; Maresco-Pennisi D.; Sargent J.; Collins L.; Edwards H.E.; Finlayson K.J. | Implementation of the Champions for Skin Integrity model to improve leg and foot ulcer care in the primary healthcare setting | 2019 | Journal of Clinical Nursing | 28 | 13-14 | 10.1111/jocn.14826 | |  |
| Parker C.N.; Van Netten J.J.; Parker T.J.; Jia L.; Corcoran H.; Garrett M.; Kwok C.F.; Nather A.; Que M.T.; Srisawasdi G.; Wraight P.; Lazzarini P.A. | Differences between national and international guidelines for the management of diabetic foot disease | 2019 | Diabetes/Metabolism Research and Reviews | 35 | 2 | 10.1002/dmrr.3101 | |  |
| Payne C.; Turner D.; Miller K. | Determinants of plantar pressures in the diabetic foot | 2002 | Journal of Diabetes and its Complications | 16 | 4 | 10.1016/S1056-8727(01)00187-8 | |  |
| Payne C.B. | Diabetes-related lower-limb amputations in Australia | 2000 | Medical Journal of Australia | 173 | 7 | 10.5694/j.1326-5377.2000.tb125685.x | |  |
| Pearson S.; Nash T.; Ireland V. | Depression symptoms in people with diabetes attending outpatient podiatry clinics for the treatment of foot ulcers | 2014 | Journal of Foot and Ankle Research | 7 | 1 | 10.1186/s13047-014-0047-4 | |  |
| Pena G.; Kuang B.; Cowled P.; Howell S.; Dawson J.; Philpot R.; Fitridge R. | Micronutrient Status in Diabetic Patients with Foot Ulcers | 2020 | Advances in Wound Care | 9 | 1 | 10.1089/wound.2019.0973 | |  |
| Pena G.; Kuang B.; Edwards S.; Cowled P.; Dawson J.; Fitridge R. | Factors Associated With Key Outcomes in Diabetes Related Foot Disease: A Prospective Observational Study | 2021 | European Journal of Vascular and Endovascular Surgery | 62 | 2 | 10.1016/j.ejvs.2021.04.002 | |  |
| Pena G.; Kuang B.; Szpak Z.; Cowled P.; Dawson J.; Fitridge R. | Evaluation of a Novel Three-Dimensional Wound Measurement Device for Assessment of Diabetic Foot Ulcers | 2020 | Advances in Wound Care | 9 | 11 | 10.1089/wound.2019.0965 | |  |
| Perrin B.M.; Allen P.; Gardner M.J.; Chappell A.; Phillips B.; Massey C.; Skinner I.; Skinner T.C. | The foot-health of people with diabetes in regional and rural Australia: Baseline results from an observational cohort study | 2019 | Journal of Foot and Ankle Research | 12 | 1 | 10.1186/s13047-019-0366-6 | |  |
| Perrin B.M.; Diacogiorgis D.; Sullivan C.; Gerrard J.; Skinner I.; Skinner T.C.; Nawaratne R.; Alahakoon D.; Kingsley M.I.C. | Habitual Physical Activity of People with or at Risk of Diabetes-Related Foot Complications | 2023 | Sensors | 23 | 13 | 10.3390/s23135822 | |  |
| Perrin B.M.; Gardner M.J.; Kennett S.R. | The foot-health of people with diabetes in a regional Australian population: A prospective clinical audit | 2012 | Journal of Foot and Ankle Research | 5 | 1 | 10.1186/1757-1146-5-6 | |  |
| Perrin B.M.; Raspovic A.; Williams C.M.; Twigg S.M.; Golledge J.; Hamilton E.J.; Crawford A.; Hargreaves C.; Van Netten J.J.; Purcell N.; Lazzarini P.A. | Establishing the national top 10 priority research questions to improve diabetes-related foot health and disease: A Delphi study of Australian stakeholders | 2021 | BMJ Open Diabetes Research and Care | 9 | 2 | 10.1136/bmjdrc-2021-002570 | |  |
| Perrin B.M.; Southon J.; McCaig J.; Skinner I.; Skinner T.C.; Kingsley M.I.C. | The Effect of Structured Exercise Compared with Education on Neuropathic Signs and Symptoms in People at Risk of Neuropathic Diabetic Foot Ulcers: A Randomized Clinical Trial | 2022 | Medicina (Lithuania) | 58 | 1 | 10.3390/medicina58010059 | |  |
| Perrin B.M.; Swerissen H.; Payne C. | The association between foot-care self efficacy beliefs and actual foot-care behaviour in people with peripheral neuropathy: A cross-sectional study | 2009 | Journal of Foot and Ankle Research | 2 | 1 | 10.1186/1757-1146-2-3 | |  |
| Perrin B.M.; Swerissen H.; Payne C.B.; Skinner T.C. | Cognitive representations of peripheral neuropathy and self-reported foot-care behaviour of people at high risk of diabetes-related foot complications | 2014 | Diabetic Medicine | 31 | 1 | 10.1111/dme.12287 | |  |
| Pitt S.; May K.; Colman P.; Wraight P. | Deficiencies in nutritional intake in patients admitted with diabetes-related foot complications | 2007 | Nutrition and Dietetics | 64 | 3 | 10.1111/j.1747-0080.2007.00096.x | |  |
| Ploderer B.; Brown R.; Da Seng L.Si.; Lazzarini P.A.; Van Netten J.J. | Promoting self-Care of diabetic foot ulcers through a mobile phone app: User-Centered design and evaluation | 2018 | JMIR Diabetes | 20 | 10 | 10.2196/10105 | |  |
| Ploderer B.; Clark D.; Brown R.; Harman J.; Lazzarini P.A.; Van Netten J.J. | Self-Monitoring Diabetes-Related Foot Ulcers with the MyFootCare App: A Mixed Methods Study | 2023 | Sensors | 23 | 5 | 10.3390/s23052547 | |  |
| Plusch D.; Penkala S.; Dickson H.G.; Malone M. | Primary care referral to multidisciplinary high risk foot services - too few, too late | 2015 | Journal of Foot and Ankle Research | 8 | 1 | 10.1186/s13047-015-0120-7 | |  |
| Poosapadi Arjunan S.; Tint A.N.; Aliahmad B.; Kumar D.K.; Shukla R.; Miller J.; Zajac J.D.; Wang G.; Viswanathan R.; Ekinci E.I. | High-Resolution Spectral Analysis Accurately Identifies the Bacterial Signature in Infected Chronic Foot Ulcers in People With Diabetes | 2018 | International Journal of Lower Extremity Wounds | 17 | 2 | 10.1177/1534734618785844 | |  |
| Quigley F.G.; Faris I.B. | Transcutaneous oxygen tension measurements in the assessment of limb ischaemia | 1991 | Clinical Physiology | 11 | 4 | 10.1111/j.1475-097X.1991.tb00660.x | |  |
| Quigley M.; Morton J.I.; Lazzarini P.A.; Zoungas S.; Shaw J.E.; Magliano D.J. | Trends in diabetes-related foot disease hospitalizations and amputations in Australia, 2010 to 2019 | 2022 | Diabetes Research and Clinical Practice | 194 |  | 10.1016/j.diabres.2022.110189 | |  |
| Quinton T.R.; Lazzarini P.A.; Boyle F.M.; Russell A.W.; Armstrong D.G. | How do Australian podiatrists manage patients with diabetes? The Australian diabetic foot management survey | 2015 | Journal of Foot and Ankle Research | 8 | 1 | 10.1186/s13047-015-0072-y | |  |
| Radzieta M.; Malone M.; Ahmad M.; Dickson H.G.; Schwarzer S.; Jensen S.O.; Lavery L.A. | Metatranscriptome sequencing identifies Escherichia are major contributors to pathogenic functions and biofilm formation in diabetes related foot osteomyelitis | 2022 | Frontiers in Microbiology | 13 |  | 10.3389/fmicb.2022.956332 | |  |
| Radzieta M.; Peters T.J.; Dickson H.G.; Cowin A.J.; Lavery L.A.; Schwarzer S.; Roberts T.; Jensen S.O.; Malone M. | A metatranscriptomic approach to explore longitudinal tissue specimens from non-healing diabetes related foot ulcers | 2022 | APMIS | 130 | 7 | 10.1111/apm.13226 | |  |
| Radzieta M.; Sadeghpour-Heravi F.; Peters T.J.; Hu H.; Vickery K.; Jeffries T.; Dickson H.G.; Schwarzer S.; Jensen S.O.; Malone M. | A multiomics approach to identify host-microbe alterations associated with infection severity in diabetic foot infections: a pilot study | 2021 | npj Biofilms and Microbiomes | 7 | 1 | 10.1038/s41522-021-00202-x | |  |
| Rasli M.H.M.; Zacharin M.R. | Foot problems and effectiveness of foot care education in children and adolescents with diabetes mellitus | 2008 | Pediatric Diabetes | 9 | 6 | 10.1111/j.1399-5448.2008.00432.x | |  |
| Raspovic A. | Gait characteristics of people with diabetes-related peripheral neuropathy, with and without a history of ulceration | 2013 | Gait and Posture | 38 | 4 | 10.1016/j.gaitpost.2013.03.009 | |  |
| Raspovic A.; Landorf K.B. | A survey of offloading practices for diabetes-related plantar neuropathic foot ulcers | 2014 | Journal of Foot and Ankle Research | 7 | 1 | 10.1186/s13047-014-0035-8 | |  |
| Raspovic A.; Landorf K.B.; Gazarek J.; Stark M. | Reduction of peak plantar pressure in people with diabetes-related peripheral neuropathy: an evaluation of the DH Pressure Relief Shoe™ | 2012 | Journal of Foot and Ankle Research | 5 | 1 | 10.1186/1757-1146-5-25 | |  |
| Raspovic A.; Newcombe L.; Lloyd J.; Dalton E. | Effect of customized insoles on vertical plantar pressures in sites of previous neuropathic ulceration in the diabetic foot | 2000 | Foot | 10 | 3 | 10.1054/foot.2000.0604 | |  |
| Raspovic A.; Waller K.; Wong W.M. | The effectiveness of felt padding for offloading diabetes-related foot ulcers, at baseline and after one week of wear | 2016 | Diabetes Research and Clinical Practice | 121 |  | 10.1016/j.diabres.2016.09.018 | |  |
| Rhou Y.J.J.; Henshaw F.R.; McGill M.J.; Twigg S.M. | Congestive heart failure presence predicts delayed healing of foot ulcers in diabetes: An audit from a multidisciplinary high-risk foot clinic | 2015 | Journal of Diabetes and its Complications | 29 | 4 | 10.1016/j.jdiacomp.2015.02.009 | |  |
| Rodrigues B.T.; Vangaveti V.N.; Malabu U.H. | Prevalence and Risk Factors for Diabetic Lower Limb Amputation: A Clinic-Based Case Control Study | 2016 | Journal of Diabetes Research | 2016 |  | 10.1155/2016/5941957 | |  |
| Rodrigues B.T.; Vangaveti V.N.; Urkude R.; Biros E.; Malabu U.H. | Prevalence and risk factors of lower limb amputations in patients with diabetic foot ulcers: A systematic review and meta-analysis | 2022 | Diabetes and Metabolic Syndrome: Clinical Research and Reviews | 16 | 2 | 10.1016/j.dsx.2022.102397 | |  |
| Rogers M.W.; Wardman D.L.; Lord S.R.; Fitzpatrick R.C. | Passive tactile sensory input improves stability during standing | 2001 | Experimental Brain Research | 137 | 1 | 10.1007/s002210000615 | |  |
| Rosi L.M.; Jones A.S.; Topliss D.J.; Bach L.A. | Demographics and outcomes of inpatients with diabetic foot ulcers treated conservatively and surgically in a metropolitan hospital network | 2021 | Diabetes Research and Clinical Practice | 175 |  | 10.1016/j.diabres.2021.108821 | |  |
| Sadler S.G.; Lanting S.M.; Searle A.T.; Spink M.J.; Chuter V.H. | Does a weight bearing equinus affect plantar pressure differently in older people with and without diabetes? A case control study | 2021 | Clinical Biomechanics | 84 |  | 10.1016/j.clinbiomech.2021.105324 | |  |
| Santamaria N.; Ogce F.; Gorelik A. | Healing rate calculation in the diabetic foot ulcer: Comparing different methods | 2012 | Wound Repair and Regeneration | 20 | 5 | 10.1111/j.1524-475X.2012.00818.x | |  |
| Saricilar E.; Gatmaitan R.; Patel K.; Yang M. | The Role of Targeted Infra-popliteal Endovascular Angioplasty to Treat Diabetic Foot Ulcers Using the Angiosome Model: A Systematic Review | 2023 | Vascular and Endovascular Review | 6 |  | 10.15420/ver.2022.08 | |  |
| Scanlon C.; Park K.; Mapletoft D.; Begg L.; Burns J. | Interrater and intrarater reliability of photoplethysmography for measuring toe blood pressure and toe-brachial index in people with diabetes mellitus | 2012 | Journal of Foot and Ankle Research | 5 | 1 | 10.1186/1757-1146-5-13 | |  |
| Schmidt A.; Schreve M.A.; Huizing E.; Del Giudice C.; Branzan D.; Ünlü Ç.; Varcoe R.L.; Ferraresi R.; Kum S. | Midterm Outcomes of Percutaneous Deep Venous Arterialization With a Dedicated System for Patients With No-Option Chronic Limb-Threatening Ischemia: The ALPS Multicenter Study | 2020 | Journal of Endovascular Therapy | 27 | 4 | 10.1177/1526602820922179 | |  |
| Schoen D.; Balchin D.; Thompson S. | Health promotion resources for Aboriginal people: Lessons learned from consultation and evaluation of diabetes foot care resources | 2010 | Health Promotion Journal of Australia | 21 | 1 | 10.1071/he10064 | |  |
| Schoen D.E.; Glance D.G.; Thompson S.C. | Clinical decision support software for diabetic foot risk stratification: Development and formative evaluation | 2015 | Journal of Foot and Ankle Research | 8 | 1 | 10.1186/s13047-015-0128-z | |  |
| Searle A.; Spink M.J.; Chuter V.H. | Weight bearing versus non-weight bearing ankle dorsiflexion measurement in people with diabetes: A cross sectional study | 2018 | BMC Musculoskeletal Disorders | 19 | 1 | 10.1186/s12891-018-2113-8 | |  |
| Searle A.; Spink M.J.; Chuter V.H. | Prevalence of ankle equinus and correlation with foot plantar pressures in people with diabetes | 2018 | Clinical Biomechanics | 60 |  | 10.1016/j.clinbiomech.2018.10.006 | |  |
| Searle A.; Spink M.J.; Ho A.; Chuter V.H. | Association between ankle equinus and plantar pressures in people with diabetes. A systematic review and meta-analysis | 2017 | Clinical Biomechanics | 43 |  | 10.1016/j.clinbiomech.2017.01.021 | |  |
| Searle A.; Spink M.J.; Oldmeadow C.; Chiu S.; Chuter V.H. | Calf muscle stretching is ineffective in increasing ankle range of motion or reducing plantar pressures in people with diabetes and ankle equinus: A randomised controlled trial | 2019 | Clinical Biomechanics | 69 |  | 10.1016/j.clinbiomech.2019.07.005 | |  |
| Searle MOsteo A.; Spink M.J.; Chuter V.H. | Validation of a weight bearing ankle equinus value in older adults with diabetes | 2018 | Journal of Foot and Ankle Research | 11 | 1 | 10.1186/s13047-018-0306-x | |  |
| Seng L.; Drovandi A.; Fernando M.E.; Golledge J. | Opinions about the most appropriate surgical management of diabetes-related foot infection: a cross-sectional survey | 2022 | Journal of Foot and Ankle Research | 15 | 1 | 10.1186/s13047-022-00523-w | |  |
| Sharma H.; Sharma S.; Krishnan A.; Yuan D.; Vangaveti V.N.; Malabu U.H.; Haleagrahara N. | The efficacy of inflammatory markers in diagnosing infected diabetic foot ulcers and diabetic foot osteomyelitis: Systematic review and meta-analysis | 2022 | PLoS ONE | 17 | 4-Apr | 10.1371/journal.pone.0267412 | |  |
| Sharma S.; Gupta R.; Compay A.; Sampson D.; Fernandez M.; Zee U.; Gary S. | Identification of diagnostic and prognostic biomarkers to improve the management of diabetes-related ulcers | 2014 | Asian Pacific Journal of Tropical Disease | 4 | 3 | 10.1016/S2222-1808(14)60521-1 | |  |
| Sheahan H.; Canning K.; Refausse N.; Kinnear E.M.; Jorgensen G.; Walsh J.R.; Lazzarini P.A. | Differences in the daily activity of patients with diabetic foot ulcers compared to controls in their free-living environments | 2017 | International Wound Journal | 14 | 6 | 10.1111/iwj.12782 | |  |
| Shiraev T.; de Boer M.; Qasabian R. | Indications for and outcomes of major lower limb amputations at a tertiary-referral centre in Australia | 2023 | Vascular | 31 | 5 | 10.1177/17085381221080811 | |  |
| Shiraev T.P.; Lipsky B.A.; Kwok T.M.Y.; Robinson D.A. | Utility of Culturing Marginal Bone in Patients Undergoing Lower Limb Amputation for Infection | 2019 | Journal of Foot and Ankle Surgery | 58 | 5 | 10.1053/j.jfas.2018.12.012 | |  |
| Shrestha M.; Ng A.; Al-Ghareeb A.; Alenazi F.; Gray R. | Association between subthreshold depression and self-care behaviors in people with type 2 diabetes: A systematic review of observational studies | 2020 | Systematic Reviews | 9 | 1 | 10.1186/s13643-020-01302-z | |  |
| Simmons D.; Bourke L.; Yau E.; Hoodless M. | Diabetes risk factors, diabetes and diabetes care in a rural Australian community | 2007 | Australian Journal of Rural Health | 15 | 5 | 10.1111/j.1440-1584.2007.00903.x | |  |
| Singh T.P.; Vangaveti V.N.; Kennedy R.L.; Malabu U.H. | Role of telehealth in diabetic foot ulcer management – A systematic review | 2016 | Australian Journal of Rural Health | 24 | 4 | 10.1111/ajr.12284 | |  |
| Siru R.; Burkhardt M.S.; Davis W.A.; Hiew J.; Manning L.; Ritter J.C.; Norman P.E.; Makepeace A.; Fegan P.G.; Bruce D.G.; Davis T.M.E.; Hamilton E.J. | Cognitive impairment in people with diabetes-related foot ulceration | 2021 | Journal of Clinical Medicine | 10 | 13 | 10.3390/jcm10132808 | |  |
| Smith L.; Plehwe W.; McGill M.; Genev N.; Yue D.K.; Turtle J.R. | Foot Bearing Pressure in Patients with Unilateral Diabetic Foot Ulcers | 1989 | Diabetic Medicine | 6 | 7 | 10.1111/j.1464-5491.1989.tb01230.x | |  |
| Smith S.E.; Miller J. | The Safety and Effectiveness of the Percutaneous Flexor Tenotomy in Healing Neuropathic Apical Toe Ulcers in the Outpatient Setting | 2020 | Foot and Ankle Specialist | 13 | 2 | 10.1177/1938640019843314 | |  |
| Sorensen L.; Molyneaux L.; Yue D.K. | Insensate versus painful diabetic neuropathy: The effects of height, gender, ethnicity and glycaemic control | 2002 | Diabetes Research and Clinical Practice | 57 | 1 | 10.1016/S0168-8227(02)00010-4 | |  |
| Stacpoole-Shea S.; Shea G.; Lavery L. | An examination of plantar pressure measurements to identify the location of diabetic forefoot ulceration | 1999 | Journal of Foot and Ankle Surgery | 38 | 2 | 10.1016/S1067-2516(99)80021-0 | |  |
| Steffen C.; O'Rourke S. | Surgical management of diabetic foot complications: The Far North Queensland profile | 1998 | Australian and New Zealand Journal of Surgery | 68 | 4 | 10.1111/j.1445-2197.1998.tb02076.x | |  |
| Stuart L.; Kimmel L.; Jolly A. | Incidence of lower limb amputation in Central Australia | 2021 | Australian Health Review | 45 | 3 | 10.1071/AH20182 | |  |
| Sung J.A.; Gurung S.; Lam T.; Yusaf S.; Vicaretti M.; Begg L.; Cheung N.W.; Padmanabhan S.; Girgis C.M. | A 'Speed-Dating' Model of Wound Care? Rapid, High-Volume Assessment of Patients with Diabetes in a Multidisciplinary Foot Wound Clinic | 2021 | Experimental and Clinical Endocrinology and Diabetes | 129 | 11 | 10.1055/a-1151-4731 | |  |
| Szwarcbard N.; Villani M.; Earnest A.; Flack J.; Andrikopoulos S.; Wischer N.; Soldatos G.; Gasevic D.; Zoungas S. | The association of smoking status with glycemic control, metabolic profile and diabetic complications– Results of the Australian National Diabetes Audit (ANDA) | 2020 | Journal of Diabetes and its Complications | 34 | 9 | 10.1016/j.jdiacomp.2020.107626 | |  |
| Tapp R.J.; Shaw J.E.; De Courten M.P.; Dunstan D.W.; Welborn T.A.; Zimmet P.Z. | Foot complications in Type 2 diabetes: An Australian population-based study | 2003 | Diabetic Medicine | 20 | 2 | 10.1046/j.1464-5491.2003.00881.x | |  |
| Tapp R.J.; Zimmet P.Z.; Harper C.A.; De Courten M.P.; Balkau B.; McCarty D.J.; Taylor H.R.; Welborn T.A.; Shaw J.E. | Diabetes care in an Australian population: Frequency of screening examinations for eye and foot complications of diabetes | 2004 | Diabetes Care | 27 | 3 | 10.2337/diacare.27.3.688 | |  |
| Tehan P.E.; Bray A.; Chuter V.H. | Non-invasive vascular assessment in the foot with diabetes: Sensitivity and specificity of the ankle brachial index, toe brachial index and continuous wave Doppler for detecting peripheral arterial disease | 2016 | Journal of Diabetes and its Complications | 30 | 1 | 10.1016/j.jdiacomp.2015.07.019 | |  |
| Tehan P.E.; Burrows T.; Hawes M.B.; Linton C.; Norbury K.; Peterson B.; Walsh A.; White D.; Chuter V.H. | Factors influencing diabetes-related foot ulcer healing in Australian adults: A prospective cohort study | 2023 | Diabetic Medicine | 40 | 1 | 10.1111/dme.14951 | |  |
| Tehan P.E.; Hawes M.B.; Hurst J.; Sebastian M.; Peterson B.J.; Chuter V.H. | Factors influencing lower extremity amputation outcomes in people with active foot ulceration in regional Australia: A retrospective cohort study | 2022 | Wound Repair and Regeneration | 30 | 1 | 10.1111/wrr.12978 | |  |
| Tehan P.E.; Hawes M.B.; Hurst J.; Sebastian M.; Peterson B.J.; Chuter V.H. | Factors influencing lower extremity amputation outcomes in people with active foot ulceration in regional Australia: A retrospective cohort study | 2022 | Wound Repair and Regeneration | 30 | 1 | 10.1111/wrr.12978 | |  |
| Thanigaimani S.; Jin H.; Ahmad U.; Anbalagan R.; Golledge J. | Comparative efficacy of growth factor therapy in healing diabetes-related foot ulcers: A network meta-analysis of randomized controlled trials | 2023 | Diabetes/Metabolism Research and Reviews | 39 | 5 | 10.1002/dmrr.3670 | |  |
| Thanigaimani S.; Singh T.; Golledge J. | Topical oxygen therapy for diabetes-related foot ulcers: A systematic review and meta-analysis | 2021 | Diabetic Medicine | 38 | 8 | 10.1111/dme.14585 | |  |
| Tran M.M.; Haley M.N. | Does exercise improve healing of diabetic foot ulcers? A systematic review | 2021 | Journal of Foot and Ankle Research | 14 | 1 | 10.1186/s13047-021-00456-w | |  |
| Van der Wall H.; Lunz D.; Stanton D.; Bruce W. | Prognostic value of 99m Tc leukocyte scintigraphy in diabetic pedal osteomyelitis | 2001 | Foot and Ankle International | 22 | 9 | 10.1177/107110070102200906 | |  |
| van Netten J.J.; Raspovic A.; Lavery L.A.; Monteiro-Soares M.; Rasmussen A.; Sacco I.C.N.; Bus S.A. | Prevention of foot ulcers in the at-risk patient with diabetes: a systematic review | 2020 | Diabetes/Metabolism Research and Reviews | 36 | S1 | 10.1002/dmrr.3270 | |  |
| van Netten J.J.; Sacco I.C.N.; Lavery L.A.; Monteiro-Soares M.; Rasmussen A.; Raspovic A.; Bus S.A. | Treatment of modifiable risk factors for foot ulceration in persons with diabetes: a systematic review | 2020 | Diabetes/Metabolism Research and Reviews | 36 | S1 | 10.1002/dmrr.3271 | |  |
| van Netten J.J.; Seng L.; Lazzarini P.A.; Warnock J.; Ploderer B. | Reasons for (non-)adherence to self-care in people with a diabetic foot ulcer | 2019 | Wound Repair and Regeneration | 27 | 5 | 10.1111/wrr.12728 | |  |
| Vangaveti V.N.; Heyes O.G.; Jhamb S.; Haleagrahara N.; Malabu U.H. | Usefulness of Procalcitonin in Diagnosing Diabetic Foot Osteomyelitis: A Pilot Study | 2021 | Wounds | 33 | 7 | 10.25270/wnds/2021.192196 | |  |
| Vangaveti V.N.; Jhamb S.; Goodall J.; Bulbrook J.; Biros E.; Malabu U.H. | Extracorporeal Shockwave Therapy (ESWT) in the Management of Diabetic Foot Ulcer: A Prospective Randomized Clinical Trial | 2023 | Journal of Foot and Ankle Surgery | 62 | 5 | 10.1053/j.jfas.2023.04.013 | |  |
| Vangaveti V.N.; Jhamb S.; Hayes O.; Goodall J.; Bulbrook J.; Robertson K.; Biros E.; Sangla K.S.; Malabu U.H. | Effects of vildagliptin on wound healing and markers of inflammation in patients with type 2 diabetic foot ulcer: a prospective, randomized, double-blind, placebo-controlled, single-center study | 2022 | Diabetology and Metabolic Syndrome | 14 | 1 | 10.1186/s13098-022-00938-2 | |  |
| Varney J.E.; Weiland T.J.; Inder W.J.; Jelinek G.A. | Effect of hospital-based telephone coaching on glycaemic control and adherence to management guidelines in type 2 diabetes, a randomised controlled trial | 2014 | Internal Medicine Journal | 44 | 9 | 10.1111/imj.12515 | |  |
| Vo U.G.; Gilfillan M.; Hamilton E.J.; Manning L.; Munshi B.; Hiew J.; Norman P.E.; Ritter J.C. | Availability and service provision of multidisciplinary diabetes foot units in Australia: a cross-sectional survey | 2021 | Journal of Foot and Ankle Research | 14 | 1 | 10.1186/s13047-021-00471-x | |  |
| Voon K.; Vo U.G.; Hand R.; Hiew J.; Ritter J.C.; Hamilton E.J.; Manning L. | Routine bacterial culture of proximal bone specimens during minor amputation in patients with diabetes-related foot infections has little clinical utility in predicting re-operation or ulcer healing | 2022 | Journal of Foot and Ankle Research | 15 | 1 | 10.1186/s13047-022-00563-2 | |  |
| Waheed F.N.M.; Vangaveti V.N.; Malabu U.H. | Ischemic heart disease and its risk factors in patients with diabetic foot ulcers: A systematic review and meta-analysis | 2022 | Diabetes and Metabolic Syndrome: Clinical Research and Reviews | 16 | 2 | 10.1016/j.dsx.2022.102414 | |  |
| Watson J.; Obersteller E.A.; Rennie L.; Whitbread C. | Diabetic foot care: developing culturally appropriate educational tools for Aboriginal and Torres Strait Islander peoples in the Northern Territory, Australia. | 2001 | The Australian journal of rural health | 9 | 3 | 10.1046/j.1440-1584.2001.00320.x | |  |
| West M.; Chuter V.; Munteanu S.; Hawke F. | Defining the gap: A systematic review of the difference in rates of diabetes-related foot complications in Aboriginal and Torres Strait Islander Australians and non-Indigenous Australians | 2017 | Journal of Foot and Ankle Research | 10 | 1 | 10.1186/s13047-017-0230-5 | |  |
| West M.; Sadler S.; Charles J.; Hawke F.; Lanting S.; Munteanu S.E.; Chuter V. | Yarning about foot care: evaluation of a foot care service for Aboriginal and Torres Strait Islander Peoples | 2022 | Journal of Foot and Ankle Research | 15 | 1 | 10.1186/s13047-022-00524-9 | |  |
| Westphal C.; Neame I.M.; Harrison J.C.; Bower V.M.; Gurr J.M. | A diabetic foot ulcer pilot study: Does silicone gel sheeting reduce the incidence of reulceration? | 2011 | Journal of the American Podiatric Medical Association | 101 | 2 | 10.7547/1010116 | |  |
| Whitmont K.; Fulcher G.; Reid I.; Xue M.; McKelvey K.; Xie Y.; Aboud M.; Ward C.; Smith M.M.; Cooper A.; March L.; Jackson C.J. | Low circulating protein C levels are associated with lower leg ulcers in patients with diabetes | 2013 | BioMed Research International | 2013 |  | 10.1155/2013/719570 | |  |
| Whitmont K.; Mckelvey K.J.; Fulcher G.; Reid I.; March L.; Xue M.; Cooper A.; Jackson C.J. | Treatment of chronic diabetic lower leg ulcers with activated protein C: A randomised placebo-controlled, double-blind pilot clinical trial | 2015 | International Wound Journal | 12 | 4 | 10.1111/iwj.12125 | |  |
| Withers R.V.; Perrin B.M.; Landorf K.B.; Raspovic A. | Offloading effects of a removable cast walker with and without modification for diabetes-related foot ulceration: a plantar pressure study | 2023 | Journal of Foot and Ankle Research | 16 | 1 | 10.1186/s13047-023-00625-z | |  |
| Wong M.; Haswell-Elkins M.; Tamwoy E.; McDermott R.; d'Abbs P. | Perspectives on clinic attendance, medication and foot-care among people with diabetes in the Torres Strait Islands and Northern Peninsula Area | 2005 | Australian Journal of Rural Health | 13 | 3 | 10.1111/j.1440-1854.2005.00678.x | |  |
| Woo K.; Dowsett C.; Costa B.; Ebohon S.; Woodmansey E.J.; Malone M. | Efficacy of topical cadexomer iodine treatment in chronic wounds: Systematic review and meta-analysis of comparative clinical trials | 2021 | International Wound Journal | 18 | 5 | 10.1111/iwj.13560 | |  |
| Woods T.-J.; Tesfay F.; Speck P.; Kaambwa B. | Economic evaluations considering costs and outcomes of diabetic foot ulcer infections: A systematic review | 2020 | PLoS ONE | 15 | 4 | 10.1371/journal.pone.0232395 | |  |
| Wu J.; Chan T.S.; Bowring G. | Functional outcomes of major lower limb amputation 1994-2006: A modern series | 2010 | Journal of Prosthetics and Orthotics | 22 | 3 | 10.1097/JPO.0b013e3181e983ac | |  |
| Xu L.; McLennan S.V.; Lo L.; Natfaji A.; Bolton T.; Liu Y.; Twigg S.M.; Yue D.K. | Bacterial load predicts healing rate in neuropathic diabetic foot ulcers | 2007 | Diabetes Care | 30 | 2 | 10.2337/dc06-1383 | |  |
| Yates C.; May K.; Hale T.; Allard B.; Rowlings N.; Freeman A.; Harrison J.; McCann J.; Wraight P. | Wound chronicity, inpatient care, and chronic kidney disease predispose to MRSA infection in diabetic foot ulcers | 2009 | Diabetes Care | 32 | 10 | 10.2337/dc09-0295 | |  |
| Yun J.-S.; Cha S.-A.; Lim T.-S.; Lee E.-Y.; Song K.-H.; Ahn Y.-B.; Yoo K.-D.; Kim J.-S.; Park Y.-M.; Ko S.-H. | Cardiovascular autonomic dysfunction predicts diabetic foot ulcers in patients with type 2 diabetes without diabetic polyneuropathy | 2016 | Medicine (United States) | 95 | 12 | 10.1097/MD.0000000000003128 | |  |
| Yuncken J.; Haines T.; Stolwyk R.J.; Williams C.M. | PoDFEd: Podiatrists and Diabetes Footcare Education Survey - How do Australian podiatrists provide diabetes education? | 2020 | Journal of Foot and Ankle Research | 13 | 1 | 10.1186/s13047-020-0376-4 | |  |
| Zhang Y.; Carter H.E.; Lazzarini P.A.; Cramb S.; Pacella R.; van Netten J.J.; Cheng Q.; Derhy P.H.; Kinnear E.M.; McPhail S.M. | Cost-effectiveness of guideline-based care provision for patients with diabetes-related foot ulcers: A modelled analysis using discrete event simulation | 2023 | Diabetic Medicine | 40 | 1 | 10.1111/dme.14961 | |  |
| Zhang Y.; Cramb S.; McPhail S.M.; Pacella R.; van Netten J.J.; Cheng Q.; Derhy P.H.; Kinnear E.M.; Lazzarini P.A. | Multiple factors predict longer and shorter time-to-ulcer-free in people with diabetes-related foot ulcers: Survival analyses of a large prospective cohort followed-up for 24-months | 2022 | Diabetes Research and Clinical Practice | 185 |  | 10.1016/j.diabres.2022.109239 | |  |
| Zhang Y.; Lazzarini P.A.; McPhail S.M.; van Netten J.J.; Armstrong D.G.; Pacella R.E. | Global disability burdens of diabetes-related lower-extremity complications in 1990 and 2016 | 2020 | Diabetes Care | 43 | 5 | 10.2337/dc19-1614 | |  |
| Zhang Y.; van Netten J.J.; Baba M.; Cheng Q.; Pacella R.; McPhail S.M.; Cramb S.; Lazzarini P.A. | Diabetes-related foot disease in Australia: a systematic review of the prevalence and incidence of risk factors, disease and amputation in Australian populations | 2021 | Journal of Foot and Ankle Research | 14 | 1 | 10.1186/s13047-021-00447-x | |  |
